# A systematic review and meta-analysis of the efficacy of alternatives to antibiotic growth promoters as strategies to reduce Salmonella in meat-type poultry (pre-harvest)

**DOI:** 10.1016/j.psj.2025.105640

**Published:** 2025-08-06

**Authors:** Amit K. Singh, Jinquan Wang, Pranita S. Patil, Deepak Subedi, Bharath Mallavarapu, Sujitha Bhumanapalli, Sasikala Vaddu, Rami A. Dalloul, Manpreet Singh, Harshavardhan Thippareddi

**Affiliations:** a Department of Agriculture and Natural Resources, Delaware State University, Dover 19901, USA; b Department of Poultry Science, Auburn University, Auburn 36849, USA; c Department of Poultry Science, University of Georgia, 120 D.W. Brooks Dr., Athens, GA 30602, USA; d Department of Food Science and Technology, University of Georgia, Athens 30602, USA; e Department of Poultry Science, University of Georgia, 109 Conner Hall, 147 Cedar Street, Athens, GA 30602, USA

**Keywords:** *Salmonella*, Systematic review, Meta-analysis, Preharvest, Interventions

## Abstract

Recent estimates indicate chicken meat products as the prominent contributing sources of foodborne salmonellosis, accounting for 18.6 % of the *Salmonella*-related illnesses. *Salmonella* in poultry processing originates at production, with the fecal-oral route being a major route of spread. The efficacy of pre-harvest interventions (probiotics, prebiotics, organic acids, essential oils, bacteriophages, vaccines, and intervention combinations) is highly variable. A systematic review and meta-analysis were conducted to evaluate the relative efficacy of pre-harvest interventions to reduce the *Salmonella* population in meat type birds. A total of 7,041 studies were identified from Web of Science, PubMed, and Google Scholar databases. After applying exclusion criteria, a total of 97 studies from 60 relevant articles were included in the meta-analysis. Due to the variability in the age and genetics of birds, rearing conditions and sample type, a standardized mean difference (SMD) based on Hedge’s G was used for the comparison and reported as the effect size. Data were analyzed using a random effect model of meta package in R version 5.1-1. Results from the random effect model were reported as A high heterogeneity (I^2^ = 80 %) was observed from the meta-analysis model. The meta-analysis revealed that most of the interventions were effective in reducing *Salmonella* population at pre-harvest with an overall SMD of -1.58 (*P≤* 0.01). Combinations of interventions showed greater reduction in *Salmonella* population in birds (SMD=-2.34, *P≤* 0.01), followed by vaccination (SMD=-2.21, *P≤* 0.01) and organic acids (SMD=-2.11, *P≤* 0.01). Probiotics showed a moderate effect in reducing *Salmonella* population in birds with SMD of -1.69 (*P≤* 0.01). Prebiotics (SMD= -0.96, *P≤* 0.01), bacteriophages (SMD= -0.81, *P≤* 0.01), and essential oils (SMD= -0.72, *P* = 0.02) were less effective compared to other interventions. The meta-analysis suggests that interventions at pre-harvest can reduce *Salmonella* populations in broilers, with combination treatments, vaccination, and organic acids being the most effective strategies. However, caution must be exercised in adopting a specific intervention, and its efficacy and safety should be evaluated prior to implementation.

## Introduction

*Salmonella* is a major foodborne pathogen causing foodborne illness outbreaks and remains the leading cause of hospitalizations and death in humans (Scallan et al., 2011; Ferrari et al., 2019; IFSAC, 2024). The U.S. Food and Drug Administration estimated that foodborne illness affects 48 million people, equivalent to more than 16 % population annually in the United States (FDA, 2022). Further, these foodborne illnesses lead to 128,000 hospitalizations and 3,000 deaths. Food security, nutrition, food safety, and the economics of production and supply are linked and inter-dependent. World Health Organization estimates provide a grim perspective of worldwide food safety and reported that 600 million people in the world fall ill from consumption of contaminated food, with an estimated 420,000 deaths and loss of US $110 billion in medical expenses and productivity each year (WHO, 2022). In the United States alone, the economic burden of foodborne illness by pathogens was estimated to be $17.6 billion in 2018 (USDA, 2023).

Poultry is one of the most affordable sources of animal protein and estimated that total worldwide poultry meat consumption will reach 15 kg per capita by 2029 (OECD/FAO, 2021). Poultry can transmit several zoonotic pathogens to humans including *Salmonella* and *Campylobacter*, major contributors to foodborne illnesses. *Salmonella* (non-typhoidal) is the second major cause of foodborne illness in the U.S. after norovirus infection, whereas it remains the top major cause for the total number of hospitalizations and deaths (CDC, 2022). *Salmonella* can cause salmonellosis (clinically characterized as enteritis), and typhoid (clinically characterized as systemic septicemia) including paratyphoid illness (Gal-Mor et al., 2014). *Salmonella* is a non-spore-forming, gram-negative rod-shaped, and flagellated bacterium of the family *Enterobacteriaceae* with several serotypes responsible for disease in humans and animals.

**Fig. 1:**
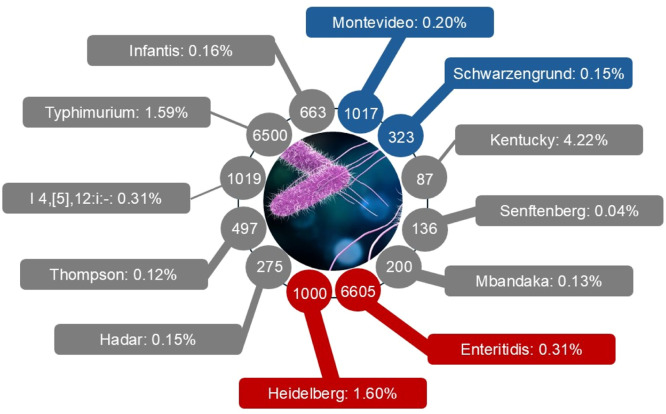
*Salmonella* serovars with their prevalence in poultry meat (rectangles) and human cases (circles) are nodes which are connected by the lines with the width represent the correlation coefficient between *Salmonella* prevalence in poultry product (raw chicken carcass, ground chicken, and retail chicken products) and human case during the period of 2002 to 2012. The color of the nodes and lines represent the statistical significance and direction of correlation: gray (*P* > 0.05), red (positive correlation, *P* ≤ 0.05), and blue (negative correlation, *P* ≤ 0.05). *Salmonella* prevalence, human cases, and correlation coefficients are summarized from Shah et al. (2017).

The *Salmonella* genus is comprised of two known species, *S. enterica* and *S. bongori* based on the surface lipopolysaccharides and flagellar antigens according to Kauffmann–White classification (Ferrari et al., 2019). The species *S. enterica* are divided into six subspecies consisting of more than 2,650 serotypes or serovars with subspecies *enterica* alone comprising over 1,550 serovars, 99 % of which are known to cause infection to both humans and animals (Gossner et al., 2016; Ferrari et al., 2019). *Salmonella* serovars are also divided into three main groups based on their ability to infect a different range of hosts. The serovars that infect a wide variety of hosts, including humans and animals, are known as generalists (e.g., *S*. Typhimurium, *S*. Enteritidis, etc.), whereas those that are restricted exclusively to certain hosts are termed as host-specific (e.g., S. *enterica* Typhi, Paratyphi, and Sendai in primates and humans, and Gallinarum and Pullorum in poultry), and the other serovars that show a preference to the particular host (e.g., *S.* Choleraesuis in pigs and *S.* Dublin in cattle) but retain the ability to occasionally affect alternate hosts are referred to as host-adapted (Silva et al., 2014; Ferrari et al., 2019; Huang et al., 2019). The generalist broad range serovars are the predominant zoonotic pathogens that cause non-typhoidal salmonellosis in humans (Fig. 1). They also infect poultry and are transmitted to humans through poultry products, a major foodborne illness.

The USDA Food Safety and Inspection Service (USDA FSIS) published the Proposed Regulatory Framework to Reduce *Salmonella* Illnesses Attributable to Poultry, a comprehensive effort to reduce *Salmonella* illnesses associated with poultry products (USDA FSIS, 2022). A key component of the framework is identifying ways to incentivize preharvest controls to reduce *Salmonella* contamination coming into the slaughterhouse. The poultry industry has adopted alternate production systems such as antibiotic-free and no-antibiotic-ever to meet the market and consumer demand for such products emanating from concerns of the emergence of multidrug-resistant microorganisms. In such a scenario, the use of non-antibiotic feed additives, vaccines, and implementation of strict biosecurity measures is essential. For the use of such non-antibiotic feed additives during pre-harvest for *Salmonella* control, USDA FSIS recommends a multi-hurdle approach, where the additive effect of products with varying modes of action could be exploited (USDA FSIS, 2022). The strategies or products that are widely used as alternatives to antibiotics are probiotics, prebiotics, organic acids, essential oils, bacteriophages, vaccines, and their combinations. These alternative products are commonly used as interventions to minimize *Salmonella* concentrations in meat-type poultry, but their relative efficacy and overall performance against *Salmonella* has not been assessed.

In this systematic review, we have focused on conducting the meta-analysis of such non-antibiotic interventions to provide insight into the consistency and effectiveness of such strategies for the control of *Salmonella* in meat-type poultry during pre-harvest production. The avian gastrointestinal tract is short, with a short passage time of less than 3-4 h on average and has a slower transit rate of digesta in cecum (Kumar et al., 2018). Thus, the emphasis has been given to comparing the effects of interventions on the population of *Salmonella* from cecal samples that represent the ideal habitat for colonization in the poultry gut.

## Materials and methods

The rationale for the review was based on the limited statistical evidence in the context of the existing knowledge regarding the effectiveness and consistency of the alternative strategies that could alone or in combination provide a sustainable substitute of antimicrobial growth promoters (AGPs) for *Salmonella* control.

### Literature search

Five authors independently carried out the literature search based on the pre-decided search strategy to include all keyword combinations that would include *Salmonella*, poultry along with the added type of poultry, and specific additives used during the last two decades of *in vivo* studies. The search database included all peer-reviewed articles, dissertations, theses, and governmental agency reports. The questions were formulated according to the well-accepted search strategy known as the population, intervention, comparison, and outcome (PICO) method. The specific questions were i) What are the prominent and effective strategies to reduce the load of *Salmonella* in poultry in the absence of antibiotic growth promoters? ii) Relative efficacy of individual strategy in reducing the *Salmonella* population in the lower gut, especially ceca of poultry? iii) Variability in the response of challenge and non-challenge (natural *Salmonella* prevalence) studies in the meat-type poultry to such intervention in terms of *Salmonella* colonization?

### Inclusion criteria for eligibility and filtering

**Fig. 2:**
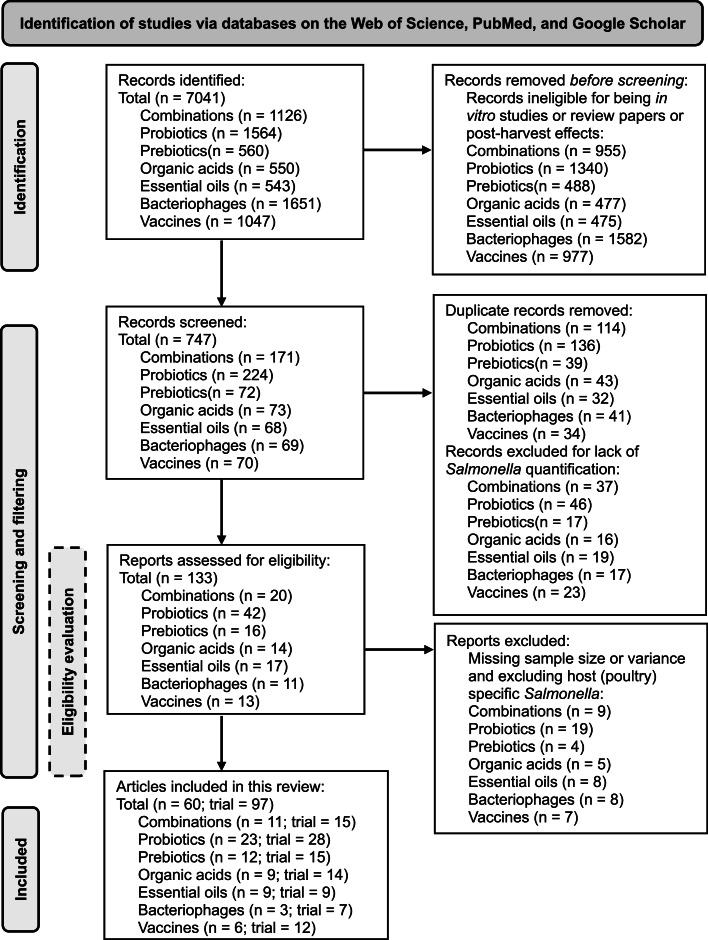
A flowchart showing the outcomes of a literature search done using the Web of Science, PubMed, and Google Scholar databases for systematic review and meta-analysis.

To address these questions, the search was divided into several categories including probiotics, prebiotics, organic acids, essential oils and phytogenics, vaccines, bacteriophages, and a combination of such interventions to make a broader coverage of each strategy. Extensive literature search was conducted in PubMed, Web of Science, and Google Scholar with a date delimitation from January 2000 to May 2023 for dietary strategies. Altogether, 7,041 database entries were identified, out of which 747 were screened, 133 were found eligible and 60 articles comprising 97 studies were included in the meta-analysis as detailed in the flowchart (Fig. 2). To keep the literature search focused and to avoid missing articles, a thorough search was conducted using titles, abstracts, and author keywords in Web of Science and PubMed, whereas separate ‘all words (*Salmonella*)’ and ‘any words [(poultry, broiler*, duck*, turkey*, and chicken*) or (additives: replaced by the names and different forms of each)]’ were used in Google Scholar. After collecting the list of searched databases from the above three sources, the list was made in Excel, and duplicates were removed. From the consolidated list, abstracts were screened to determine if the studies were acceptable for the scope of the meta-analysis based on the aforementioned questions. Those articles with no *in vivo* or poultry experiments were excluded as they did not meet the first criterion. The remaining filtered studies were retained for further steps. The full articles were then downloaded and reviewed in detail and those studies that declared sample size/experimental unit (n) of birds and *Salmonella* population means along with error information were screened to be qualified for data collection. Those studies that reported sample size of birds taken from the same rearing unit in studies that were provided products through feed were also excluded from data extraction as confounding spurious association would occur due to the lack of independence of subjects. However, in the experiments that researchers applied intervention via feed or water and reared the birds together in one unit but collected samples separately were included in the meta-analysis. The sampled birds number is regarded as the sample size (n) in this case. The scores were not assigned to any study as different challenge serotypes and classes and inclusion rates of products were used in different studies and incorporating such scores would add complexity to the interpretation of the meta-analysis (Wang et al., 2023).

### Data extraction

The data were collected from the tables as presented and Image J software was used to extract the mean and error values from the graphs. In the studies that included *Salmonella* population at different time points, the final or the longest duration was considered for the comparison. The samples from the live birds were further divided into ileal, cecal, cloacal, and fecal, and the emphasis was on including the cecal *Salmonella* population when possible. If the study presented a standard error (SE) or standard error of the mean, then the standard deviation (SD) was calculated as $SD=SE\;\times \sqrt{n}$, where n is the sample size. In one instance, the author presented the mean population along with the range and the SD was estimated as one-fourth of the range. All extracted information was stored in Microsoft Excel spreadsheet. The quality scores were not assigned to the extracted data in the meta-analysis to prevent any inadvertent selection bias (Stone et al., 2019). In studies that reported different doses of interventions, the data were combined in one group using formula reported in Chapter 7.7.3.8 and Table 7.7a of Cochrane Handbook for Systematic Reviews of Interventions (Antes et al., 2019).

### Data analyses

The collected data were used to conduct a meta-analysis and the results are reported in both tables and forest plots in accordance with the Preferred Reporting Items for Systematic Review and Meta-Analyses (PRISMA 2020) guidelines. The meta-analysis was conducted using the meta package (Schwarzer, 2007) in R software 4.0.2 (R Core Team, 2019), and the output was presented as forest plots for visualization. Because several studies included in this meta-analysis did not include the same class of products, used different levels and dosages of products, used different populations of *Salmonella* challenge, and quantified *Salmonella* populations at different ages in different breeds of meat type birds, the use of the mean difference in the meta-analysis would not be a correct measure of effect size. Therefore, a standardized mean difference (SMD) based on Hedge’s G and the calculated variance for Hedge’s G was employed in the present meta-analysis to produce the forest plots. The SMD presents the effect size as the reduction of *Salmonella* population that is visible in a scaled number and indicates the times of the pooled standard deviations among those experiments. The SMD in the forest plot is displayed with a 95 % confidence interval (CI) along with the tau-squared (τ^2^) variance that describes the variance between the studies (Higgins et al., 2019) and I^2^ as the measure of heterogeneity. The value of I^2^ up to 40 % was considered not important, 40–75 % was considered moderate or substantial, and beyond 75 % was considered considerable (Deeks et al., 2019).

### Test for publication bias

The number of studies included under different additive types in this meta-analysis was not sufficient (n≥10) for group comparisons and had a moderate or substantial heterogeneity ($I^{2}>40\%)$ that created limitations for statistically quantifying publication bias through funnel plots (Ioannidis and Trikalinos, 2007).

## Results and discussion

**Fig. 3:**
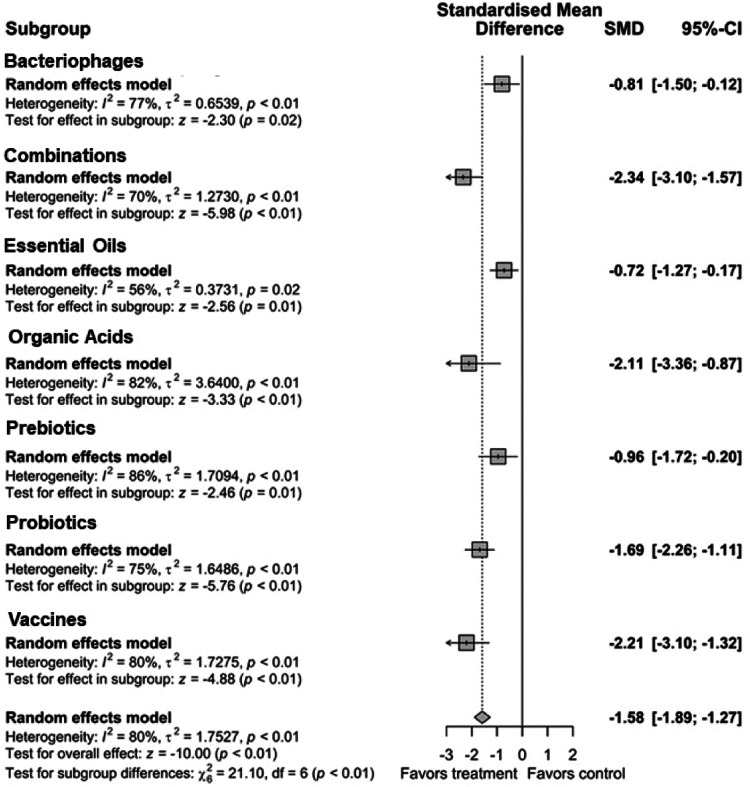
Subgroup summary forest plot of meta-analysis results of all additives interventions to control *Salmonella* colonization in meat-type poultry. Abbreviations: CI, confidence interval; τ^2^, tau-squared; χ^2^, chi-squared; df, degrees of freedom; I^2^, I-squared statistic. The vertical line at the value of zero (0) in the scale of the forest plot is the line of no effect. The hyphen (-) represents a negative standardized mean difference (SMD) effect size and corresponds to a higher *Salmonella* population in the control compared with the treatment group. The gray square boxes represent the point estimates of individual studies with a 95 % confidence interval horizontal lines extending on both sides. The mid-point of the gray boxes is the mean effect estimate and the area of the boxes corresponds to the weight of each study. The diamond at the bottom represents the summary estimate and confidence interval of all studies combined. The points on the vertical angle of the diamond represent the overall effect and the width of the diamond represents the 95 % confidence interval.

A thorough review and detailed meta-analysis performed by Wang et al. (2023) identified sources for *Salmonella* colonization in poultry such as litter, hatchery, feed, chicks, poultry house environment, water, etc. in broiler production. The authors reported hatchery (48.5 %) as a major source of colonization, followed by litter (25.4 %), and feces (16.3 %) as the other notable sources of *Salmonella* in poultry. *Salmonella* can colonize the poultry gut through various routes, ranging from respiratory to cloacal, but the fecal-oral route remains the primary route of colonization in poultry. Antibiotics at subtherapeutic concentrations have been used as growth promoters as the preferred choice for controlling *Salmonella* in meat-type poultry before restricting their use due to concern of antibiotic resistance (Van Immerseel et al., 2002; Punchihewage-Don et al., 2022). In the post-antibiotic era of poultry production, several intervention strategies have been reported for *Salmonella* control in poultry flocks during the pre-harvest period (Byrd et al., 2003; Vandeplas et al., 2010; Ruvalcaba-Gómez et al., 2022). Major intervention strategies include one or more of the following: biosecurity, vaccination, feed/water-based additives such as probiotics, prebiotics, organic acids, essential oils, and bacteriophages (Fig. 3). Those interventions are considered safe for human and animal consumption and categorized as generally recognized as safe (GRAS; FDA, 2004) in the US. Incorporating pre-harvest interventions, the *Salmonella* load in or on the birds could be reduced and the concentration of *Salmonella* entering the processing phase at post-harvest will be limited to the minimum possible, reducing the risk of contamination and consequently, the foodborne illness related to *Salmonella*. A total of 7,041 articles were identified (combinations = 1,126, probiotics = 1,564, prebiotic = 560, organic acids = 550, essential oils = 543, bacteriophages = 1,651, and vaccines = 1,047), and after screening for relevant articles based on research on birds during pre-harvest, *Salmonella* quantification, availability of sample size and variance, 97 indexes from 55 relevant peer reviewed publications were included in the meta-analysis (combinations = 15, probiotics = 27, prebiotic = 16, organic acids = 11, essential oils = 9, bacteriophages = 7, and vaccines = 12).

### Salmonella control strategies

Biosecurity is an indispensable strategy to control *Salmonella* spread and colonization in poultry production. The scope of biosecurity is broad, and it incorporates management practices that restrict the movement of people and other disease-transmitting vectors. It could include isolation and quarantine as biocontainment practices, as well as eliminating and/or reducing the pathogen through disinfection, filtration, and deactivation (Totton et al., 2012). It remains the basic underlying line of protection for birds from potential pathogens. However, the implementation of such strategies is dependent on the commitment and awareness of the producers and the implemented protocols to perform disinfection and maintenance of the zone of separation. The effectiveness of biosecurity measures in different types of production practices has been discussed earlier (Samanta et al. (2018) Velasquez et al. (2018)). Literature containing quantification of the *Salmonella* populations as colony forming units in control and experimental (biosecurity practice) conditions was limited. Calculation of SMD and comparison with other interventions was not possible. Therefore, meta-analysis of biosecurity as an intervention was not within the scope of this review. Readers are referred to a well-presented systematic review by Totton et al. (2012) where the authors compared the effects of biosecurity in terms of *Salmonella* prevalence in broilers expressed as odds ratio effect size in the meta-analysis.

### Effects of non-antibiotic intervention strategies on birds

**Fig. 4:**
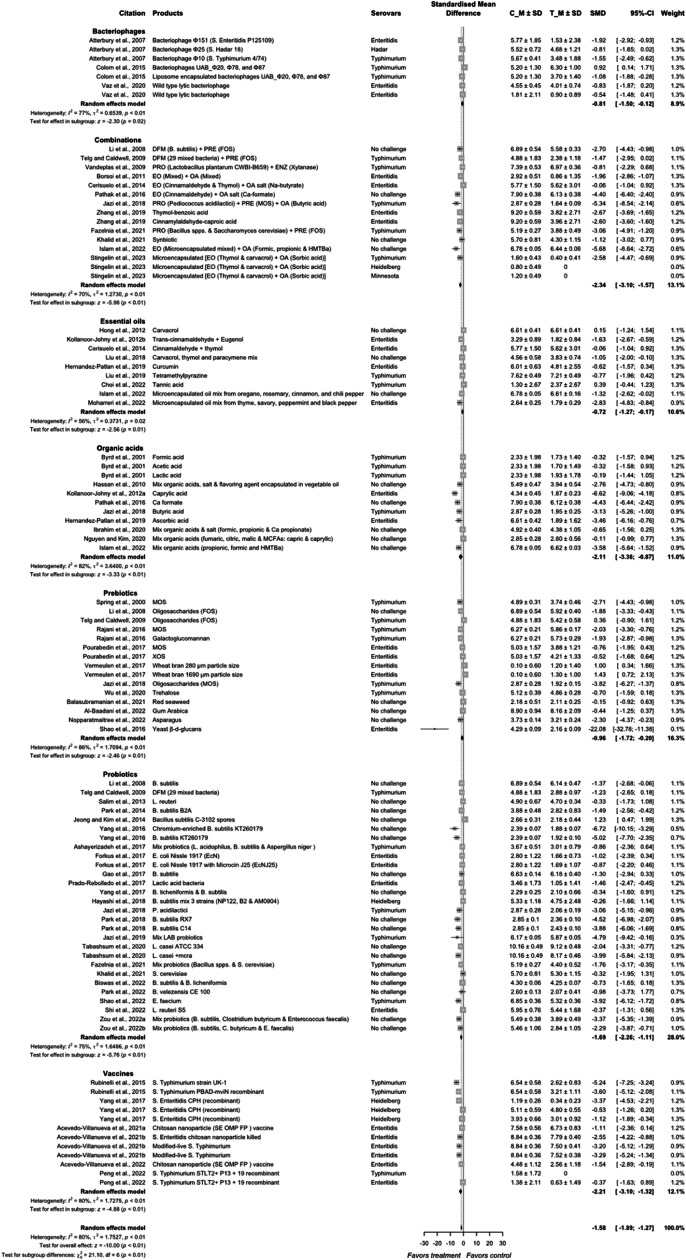
Summary forest plot of all types of additives used to control *Salmonella* colonization in meat-type poultry. Abbreviations: CI, confidence interval; τ^2^, tau-squared; χ^2^, chi-squared; df, degrees of freedom; I^2^, I-squared statistic. The vertical line at the value of zero (0) in the scale of the forest plot is the line of no effect. The hyphen (-) represents a negative standardized mean difference (SMD) effect size and corresponds to a higher *Salmonella* population in the control compared with the treatment group. The gray square boxes represent the point estimates of individual studies with a 95 % confidence interval horizontal lines extending on both sides. The mid-point of the gray boxes is the mean effect estimate and the area of the boxes corresponds to the weight of each study. The diamond at the bottom represents the summary estimate and confidence interval of all studies combined. The points on the vertical angle of the diamond represent the overall effect and the width of the diamond represents the 95 % confidence interval.

A random effects model of the meta-analysis was conducted to evaluate the effects of alternative strategies collectively named additives to control *Salmonella* in meat-type poultry and the results are presented in Fig. 4. From the overall random effects model of subgroups of additives, the additives reduced *Salmonella* population in the intestinal digesta, or excreta of birds compared with control groups (*P* < 0.01). Of note, the effect size was calculated by subtracting the mean value of control from the treatment groups and standardized with respect to their pooled standard deviation as Hedges’s G. Therefore, the negative value of SMD represents the positive impact of the additives as these interventions reduced *Salmonella* concentration in the birds.

The overall SMD of −1.58 states that on average these strategies would reduce the *Salmonella* concentration in a flock of birds by 1.58 times the measured standard deviation on a log_10_ scale. For example, if the *Salmonella* count in a control bird is 4.5 ± 0.7 log_10_ CFU/g (estimated from meta-analysis data), these additives would reduce the mean *Salmonella* concentration to 4.5 − (0.7 × 1.58), i.e., 3.4 log_10_ CFU/g. However, there was a difference in the effect size of the individual additive groups tested (*P* < 0.01); intervention combinations (combinations of additives/interventions) showed the highest reduction in *Salmonella* concentration in birds (SMD = −2.34, *P* < 0.01), followed by the vaccination (SMD = −2.21, *P* < 0.01) and organic acids (SMD = −2.11, *P* < 0.01). Additionally, probiotics showed a moderate effect in reducing the *Salmonella* population in birds with SMD of −1.69 (*P* < 0.01). Prebiotics (SMD = −0.96, *P* < 0.01), bacteriophages (SMD = −0.81, *P* < 0.01), and essential oils (SMD = −0.72, *P* = 0.02) were least effective compared to other interventions.

### Organic acids

Organic acids are products of microbial fermentation when oxygen is not available as a terminal electron acceptor, contain carboxyl groups (-COOH) and are often classified based on the number of carbon atoms on the chain as short chain fatty acids (SCFA, C1-C6), medium chain fatty acids (MCFA, C7-C10), and long chain fatty acids (LCFA, ≥11; Van Immerseel et al., 2006). Among the groups, SCFA (formic acid, acetic acid, propionic acid, and butyric acid) and MCFA (caprylic and capric acids) are used as antimicrobials in poultry production (Dittoe et al., 2018). The use of organic acids in live production primarily relies on their antimicrobial activity, through lowering the pH of the gastrointestinal tract and inhibiting pathogenic bacteria (Van Immerseel et al., 2006). Beyond their antimicrobial activity, organic acids were also reported to enhance nutrient utilization and animal performance by serving as energy sources in intestinal epithelial cells (Qaisrani et al., 2015), stimulating endogenous enzyme secretion (Jazi et al., 2019), and promoting gut development (Liu et al., 2017). In this systematic review and meta-analysis, the focus is on the antimicrobial effects of organic acids on reducing *Salmonella* colonization and population in meat-type birds at pre-harvest.

The SMD of organic acids on *Salmonella* reduction in meat type poultry is −2.11 (95 % CI = −3.36, −0.87) with heterogeneity between studies of 82 % (*P* < 0.01; Fig. 5a, Fig. 5b). The SMDs of the non-challenge group (−2.12; *P* < 0.01) and challenge group (−2.15; *P* < 0.01) are similar (*P* = 0.98) with a heterogeneity of 84 %. Organic acids are commonly supplemented in the feed or drinking water in pure forms or as mixtures. In feed, organic acids are often provided in its salt form and encapsulated in fatty acids to reduce the irritation and odor during handling and feed mixing as well as to deliver the organic acids to the hind gut (Hassan et al., 2010; Jazi et al., 2018; Nguyen and Kim., 2020; Islam et al., 2022).

**Fig. 5a:**
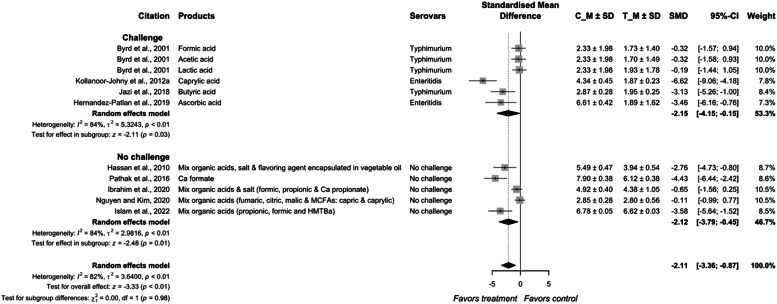
Forest plot of organic acids additives used to control *Salmonella* colonization in meat-type poultry. Abbreviations: CI, confidence interval; τ^2^, tau-squared; χ^2^, chi-squared; df, degrees of freedom; I^2^, I-squared statistic. The vertical line at the value of zero (0) in the scale of the forest plot is the line of no effect. The hyphen (-) represents a negative standardized mean difference (SMD) effect size and corresponds to a higher *Salmonella* population in the control compared with the treatment group. The gray square boxes represent the point estimates of individual studies with a 95 % confidence interval horizontal lines extending on both sides. The mid-point of the gray boxes is the mean effect estimate and the area of the boxes corresponds to the weight of each study. The diamond at the bottom represents the summary estimate and confidence interval of all studies combined. The points on the vertical angle of the diamond represent the overall effect and the width of the diamond represents the 95 % confidence interval.

**Fig. 5b:**
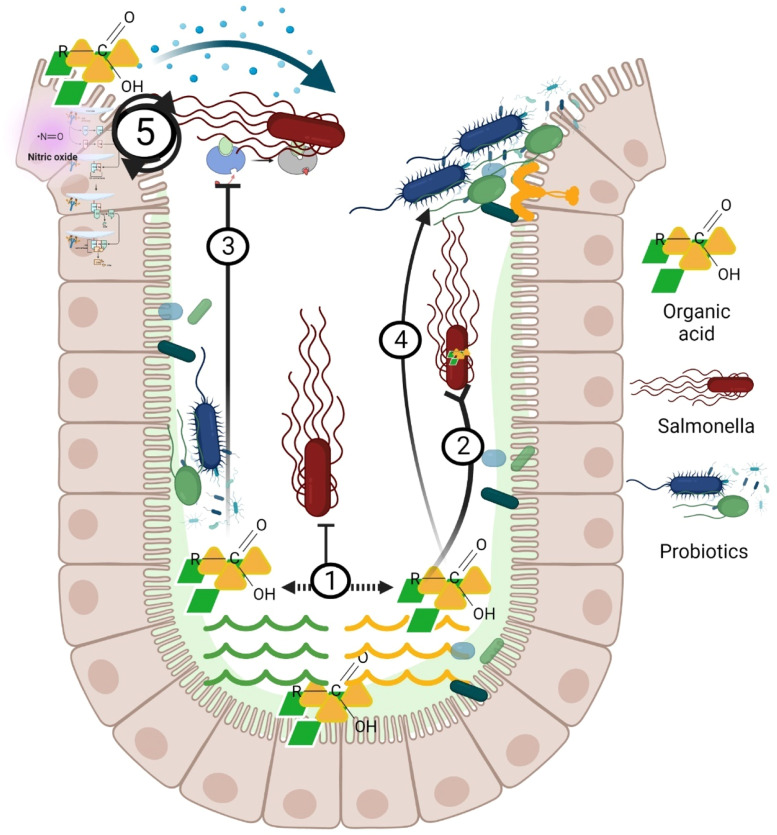
Mechanism of action organic acids approach inhibits *Salmonella* colonization in chicken gastrointestinal tract. ① Luminal pH reduction causing unfavorable environment for *Salmonella* colonization and multiplication. ② Internalization and dissociation causing disruption of bacterial cell. ③ Inhibition of bacterial enzymes. ④ Modulation of gut microbiota. ⑤ Immunomodulation such as regulation of cytokines, activation of complement system and the production of nitric oxide to defend against *Salmonella.*

Organic acids remaining in the undissociated form below their pKa, can penetrate microbial cells, thus reducing intracellular pH and altering cell membrane permeability (Li et al., 2012). Additionally, organic acids can lower the gastrointestinal tract pH and thus, stimulate the conversion of pepsinogen to pepsin (Suiryanrayna and Ramana, 2015). Historically, the use of organic acids in feed or water can lower the pH in the crop and gizzard of the bird, reducing microbial growth and the potential for horizontal transmission; however, *Salmonella* population and prevalence are not affected in the ceca (Byrd et al., 2001; Van Immerseel et al., 2006). Organic acids can increase the *Lactobacillus* and *Bifidobacterium* population in the ceca and excreta of birds (Jazi et., 2018; Ibrahim et al., 2020; Nguyen and Kim, 2020). However, the efficacy of organic acids was inconsistent across the studies, possibly due to the carbon chain length (Van Immerseel et al., 2004; Lacroix-Lamandé et al., 2023). Fatty acids with more carbons such as caproic acid, butyric acid and propionic acid decrease the invasion of *Salmonella* Enteritidis in epithelial cells, while acetic acid and formic acid increased the invasion (Van Immerseel et al., 2003 and Kollanoor-Johny et al., 2012a).

Microorganisms, especially *Salmonella* can exhibit resistance to organic acids through acid shock and other similar responses, to survive acidic environments (Ricke, 2003; Dittoe et al., 2018). *Salmonella* can also express increased resistance and survive in macrophages when it is pre-incubated with SCFA (Kwon and Ricke, 1998; Calhoun and Kwon, 2010). Besides, *Salmonella* is also capable of catabolizing SCFA for re-entry into the tricarboxylic acid cycle and producing fermentation acids under anaerobic conditions providing self-protection from SCFA as it is similar to their own end products (Foley et al., 2013). Van Immerseel et al. (2006) reported that caprylic and capric acids are more effective against *Salmonella* than SCFA due to its bactericidal effects.

The potential detrimental effect on lactic acid bacteria accounts for the main challenge for employing organic acids in poultry production (Knarreborg et al., 2002). Early studies have indicated that supplementation of formic acid and propionic acid not only reduced the *Salmonella* population in the birds’ crop and food slurry, but also reduced *Lactobacillus* and lactic acid bacteria populations (Thompson and Hinton, 1997). Another challenge related to the use of organic acids is that little if any dietary organic acids reach the lower digestive tract and the ceca (Hume et al., 1993). Encapsulation technology protects the degradation and absorbance of organic acids in the foregut, allowing them to be delivered further down the digestive tract where they can exert their beneficial effects on controlling *Salmonella* at pre-harvest (Hassan et al., 2010; Jazi et al., 2018; Liu et al., 2019). Research is needed to validate the efficacy of different organic acids, doses, targeted delivery systems, and application time for reducing *Salmonella* population in bird gut.

### Probiotics

Probiotics or direct-fed microbials (DFM) are live microorganisms, mainly as the endospore forming or acid resistant bacteria that can survive the upper gut, are expected to provide a range of benefits to the host, especially through the competitive exclusion of pathogens and modulation of the host immune systems (Jha et al., 2020). *Lactobacillus, Bacillus, Enterococcus, Saccharomyces*, and *Bifidobacterium* genera are among the most used probiotics in animal nutrition (Gaggìa et al., 2010; Markowiak and Śliżewska, 2018). Probiotics act through various actions, mainly via regulating intestinal homeostasis (Liu et al., 2023), maintaining the gut integrity of gastrointestinal barrier (Meyer et al., 2019), and regulating immunity (Keerqin et al., 2021) thus improving gut health, nutrient absorption, and performance (Gaggìa et al., 2010).

The overall effect size of *Salmonella* reduction in meat-type poultry through the application of probiotics was estimated to be −1.69 (95 % CI = −2.26; −1.11) with a heterogeneity of 75 % among different studies (*P* < 0.01; Fig. 6a, Fig. 6b). The effect of probiotics was different between challenge (SMD = −1.26) and no-challenge (SMD = −1.93) studies (*P* < 0.01). The heterogeneity of no-challenge studies was 82 % (*P* < 0.01) and that for challenge studies was 42 % (*P* = 0.03).

**Fig. 6a:**
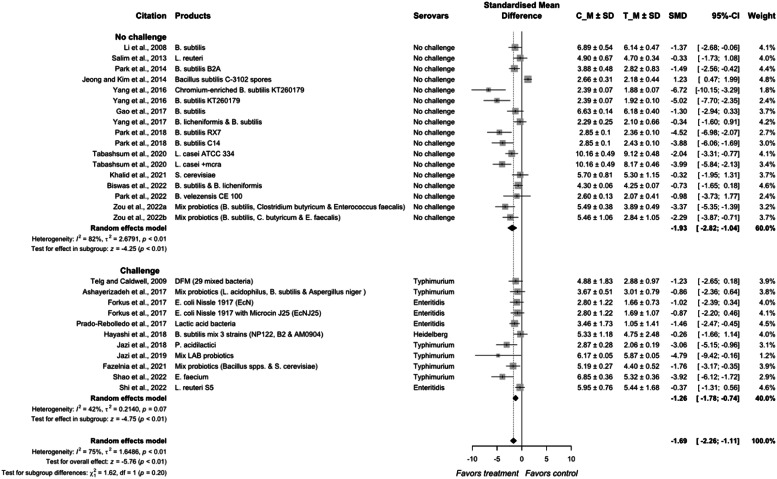
Forest plot of probiotics additives used to control *Salmonella* colonization in meat-type poultry. Abbreviations: CI, confidence interval; τ^2^, tau-squared; χ^2^, chi-squared; df, degrees of freedom; I^2^, I-squared statistic. The vertical line at the value of zero (0) in the scale of the forest plot is the line of no effect. The hyphen (-) represents a negative standardized mean difference (SMD) effect size and corresponds to a higher *Salmonella* population in the control compared with the treatment group. The gray square boxes represent the point estimates of individual studies with a 95 % confidence interval horizontal lines extending on both sides. The mid-point of the gray boxes is the mean effect estimate and the area of the boxes corresponds to the weight of each study. The diamond at the bottom represents the summary estimate and confidence interval of all studies combined. The points on the vertical angle of the diamond represent the overall effect and the width of the diamond represents the 95 % confidence interval.

**Fig. 6b:**
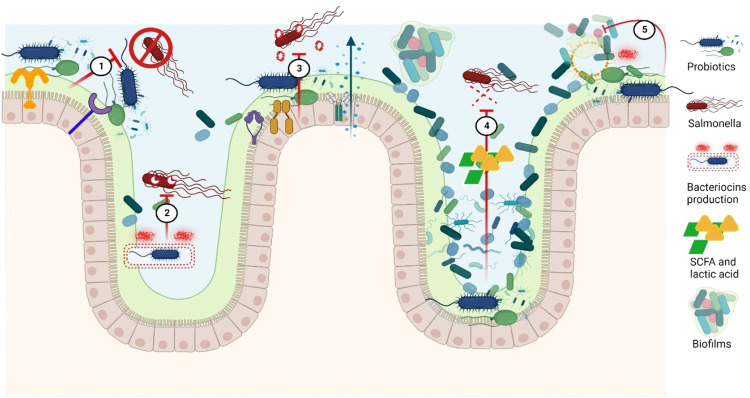
Mechanism of action probiotics approach inhibits *Salmonella* colonization in chicken gastrointestinal tract. ① Competitive exclusion by Probiotics for receptor binding and nutrients with *Salmonella*. ② Bacteriocins such as salmocins that are cationic peptides capable of killing *Salmonella* by pore formation. ③ Immunomodulation and change in gene expression of immunoglobulins, cytokines and antioxidants. ④ Acidification of gut by increased fermentation metabolites such as lactic acid and SCFA. ⑤ Disruption/eradication of extracellular polymeric substances (biofilms) of *Salmonella* through surfactants, bacteriocins and other metabolites.

Probiotics could be provided to the birds through feed or water either as individual strains or a mixture of several species. Probiotics are the most evaluated among all the interventions with a total of 28 indexes identified from the literature (12 challenge and 16 no-challenge studies). *Bacillus* subtilis is the most abundantly tested strain in poultry live production with 15 indexes due to their effect in promoting gut health and as an extension of use in humans (Saengkerdsub and Ricke, 2014). Another advantage of *Bacillus* spp. is its ability to form spores, which are highly resistant to physical and chemical changes encountered during feed manufacturing (e.g. pelleting) and within the gastrointestinal tract (Popov et al., 2021). This results in higher viability and efficiency of *Bacillus* spp. as probiotics in the hindgut.

*Salmonella* is known to be an intracellular bacterium and probiotics that reduce *Salmonella* likely do so by apoptosis mechanism (Tellez et al. (2012). In other studies, treatment improved the probiotic effect numerically, yet these studies were not significant in the meta-analysis due to fewer replications used and reported large standard deviations. One of the reasons for the low efficacy of several probiotics in different studies is that these exogenous probiotics may not colonize well in the intestinal tract of poultry compared with *Salmonella* serovars (Forkus et al., 2017). A few studies reported that the effect of probiotics on *Salmonella* colonization is variable and depended on the stress level of the birds prior *Salmonella* exposure, temperature, environment of rearing and biosecurity status of the farm (Bailey et al., 1991; Khan and Naz, 2013; Borsoi et al., 2015). Being live cells, probiotics vary in their ability to establish and proliferate in competition with *Salmonella* serovars, and their impact on poultry health is dependent on the interaction between local immunity and gut microbiota diversity of the birds (Wang et al., 2018; Ramlucken et al., 2020). Thus, it is necessary to identify the specific conditions that would make the probiotics functional with maximal efficacy.

### Prebiotics

Prebiotics such as complex sugars, oligosaccharides, glucans, and resistant starch, etc., are dietary ingredients that are not digested by the host but are selectively fermented and utilized by the beneficial microbes and provide health benefits to the host (Gibson et al., 2010). Compounds like mannan-oligosaccharides (MOS), fructooligosaccharides (FOS), xylooligosaccharide (XOS), galactooligosaccharides (GOS), and inulin are commonly used as prebiotics in broilers (Ricke, 2018). Most prebiotics are derived from the bakery or distiller fermentation process of yeast. Prebiotics are reported to modulate the immune system, provide binding sites for bacteria, increase the fermentation metabolites such as SCFA, and reduce pathogen loads such as *Salmonella* (Micciche et al., 2018; Singh and Kim, 2021; Wang et al., 2025).

The overall model effect size for the reduction of *Salmonella* by prebiotics was −0.96 (95 % CI = −1.72; −0.20) with a heterogeneity of 86 % between the studies (*P* < 0.01; Fig. 7a, Fig. 7b). Studies from challenge models had an estimated reduction in *Salmonella* of SMD = −1.01 (95 % CI = −2.07; 0.04) with heterogeneity of 89 % (*P* < 0.01) and had an SMD = −0.90 (95 % CI = −1.83; 0.03) with heterogeneity of 58 % from no-challenge studies. No difference was found in the outcomes of challenge and non-challenge studies (*P* = 0.88).

**Fig. 7a:**
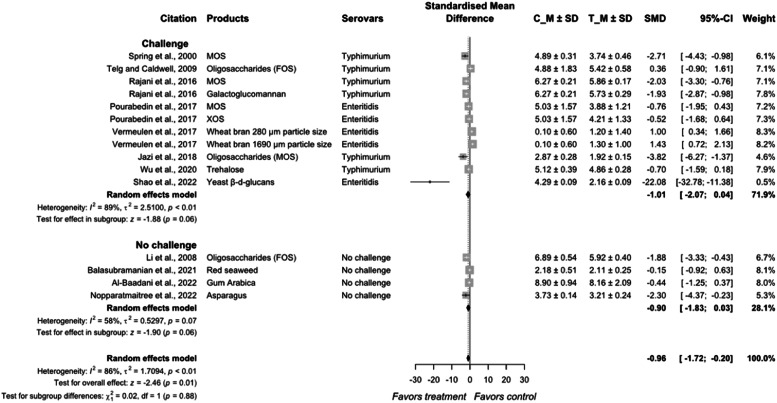
Forest plot of prebiotics additives used to control *Salmonella* colonization in meat-type poultry. Abbreviations: CI, confidence interval; τ^2^, tau-squared; χ^2^, chi-squared; df, degrees of freedom; I^2^, I-squared statistic. The vertical line at the value of zero (0) in the scale of the forest plot is the line of no effect. The hyphen (-) represents a negative standardized mean difference (SMD) effect size and corresponds to a higher *Salmonella* population in the control compared with the treatment group. The gray square boxes represent the point estimates of individual studies with a 95 % confidence interval horizontal lines extending on both sides. The mid-point of the gray boxes is the mean effect estimate and the area of the boxes corresponds to the weight of each study. The diamond at the bottom represents the summary estimate and confidence interval of all studies combined. The points on the vertical angle of the diamond represent the overall effect and the width of the diamond represents the 95 % confidence interval.

**Fig. 7b:**
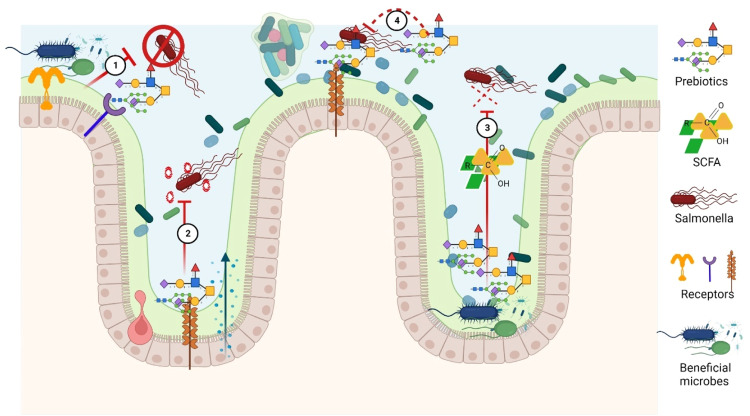
Mechanism of action probiotics approach inhibits *Salmonella* colonization in chicken gastrointestinal tract. ① Promote beneficial bacteria for competitive exclusion of *Salmonella*. ② Immunomodulation and regulates gene expression of adaptive immune cells and cytokines. ③ Acidification of gut by increased fermentation metabolites such as lactic acid and SCFA. ④ Prebiotics like mannan-oligosaccharides can bind to *Salmonella* and prevent their adhesion to the gut wall.

Most of the oligosaccharides evaluated showed a very limited effect of less than 1 log CFU/g in reducing *Salmonella* population in the ceca. The only study that reported an effective reduction in *Salmonella* population was Shao et al. (2016), who reported a yeast β-d-glucan from dried cell wall of *Saccharomyces cerevisiae*. The lower *Salmonella* population was reportedly due to the synthesis of endogenous antimicrobial peptides (cathelicidins and LEAP-2) and reinforcing humoral immunity to promote innate hose defense.

Conversely, Telg and Caldwell (2009) reported that dietary incorporation of FOS resulted in an increase in *Salmonella* population in the ceca of birds during the 8-d study. Similarly, Vermeulen et al. (2017) reported that wheat bran inclusion reduced *Salmonella* population during the 14-d period, but not later at the harvest age of d 42 where *Salmonella* population in the ceca increased. Corn-based diets can have lower levels of *Salmonella* in the ceca, liver and spleen, potentially due to the differential non-starch polysaccharides in cereal grains (Teirlynck et al., 2009). Vandeplas et al. (2009) reported that supplementation of xylanase to a wheat-based diet reduced *Salmonella* population in the excreta, while addition of *Lactobacillus* to the enzyme did not show any additive effect in reducing *Salmonella* population. Thus, the effect of prebiotics on *Salmonella* colonization in poultry should be interpreted with caution. Notably, FOS can show adverse effects by increasing intestinal permeability thus exhibiting inconsistent results as their utilization depends on several factors including degree of polymerization, composition of the basal diet, host stress, and gut microbiome composition (Micciche et al., 2018). The microbiome of the chicken gut is age-dependent and becomes more complex in older birds compared with young chicks and the bacteria that do not possess prebiotic fermenting enzymes could also benefit from the hydrolyzed products from other fermenters (Ballou et al., 2016; Ricke et al., 2020). Additionally, *Salmonella* could tolerate well the effects of fermentation metabolites of prebiotics in an anaerobic environment that is established when oxygen is consumed by the prolific growth of aerobes as the chicks grow older (Foley et al., 2013; Aruwa et al., 2021).

### Essential oils

Essential oils are concentrated, aromatic oily liquids extracted from plant materials (Burt, 2004). In addition to essential oils, this systematic review and meta-analysis also includes other plant-derived compounds such as phytobiotics, phytogenics, and botanicals. These substances, primarily secondary metabolites, play a crucial role in protecting plants from predators and microbial pathogens through their biocidal properties and ability to repel herbivores (Bassole and Juliani., 2012). Based on their components, essential oils can be grouped into: (i) terpene compounds, and (ii) aroma compounds (Bakkali et al., 2008). The antimicrobial and other biological activities of essential oils are directly linked to their bioactive volatile components (Mahmoud and Croteau, 2002). The mechanisms of the antimicrobial activity of essential oils have been associated with the phenolic and hydrophobic nature to disrupt cell membrane permeability and integrity, interfere with ATP generation systems and proton move force (Burt, 2004; Friedly et al., 2009; Bajpai et al., 2012, Micciche et al., 2019).

Essential oils used in animal production are often a variable mixture of terpenoids, and low molecular weight hydrocarbons such as thymol, carvacrol, eugenol, cinnamaldehyde, phenols, or others. The proposed mechanism of action of essential oils is their interaction with the lipid bilayer of *Salmonella* due to its hydrophobicity that can lead to cell lysis and leakage of cellular contents (Abd El-Hack et al., 2022), as well as reducing their pathogenicity and biofilm formation by inhibiting quorum-sensing (Szabó et al., 2010).

Results from the current systematic review and meta-analysis showed that the overall effect size of essential oils on *Salmonella* reduction in meat-type poultry was −0.72 (95 % CI = −1.27; −0.17) with heterogeneity between individual studies of 56 % (*P* < 0.01; Fig. 8a, Fig. 8b). The effects of essential oil in the no-challenge groups were −0.83 with heterogeneity of 25 % and had no difference in *Salmonella* reduction among studies (*P* = 0.03). The effect of essential oils in the challenge groups had an SMD of −0.73 (95 % CI = −1.51; 0.05) and a heterogeneity of 61 %. The overall effects of the non-challenge groups were not different from the challenge groups (*P* = 0.85).

**Fig. 8a:**
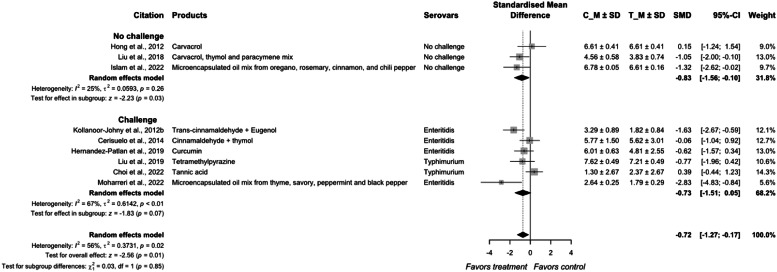
Forest plot of essential oils additives used to control *Salmonella* colonization in meat-type poultry. Abbreviations: CI, confidence interval; τ^2^, tau-squared; χ^2^, chi-squared; df, degrees of freedom; I^2^, I-squared statistic. The vertical line at the value of zero (0) in the scale of the forest plot is the line of no effect. The hyphen (-) represents a negative standardized mean difference (SMD) effect size and corresponds to a higher *Salmonella* population in the control compared with the treatment group. The gray square boxes represent the point estimates of individual studies with a 95 % confidence interval horizontal lines extending on both sides. The mid-point of the gray boxes is the mean effect estimate and the area of the boxes corresponds to the weight of each study. The diamond at the bottom represents the summary estimate and confidence interval of all studies combined. The points on the vertical angle of the diamond represent the overall effect and the width of the diamond represents the 95 % confidence interval.

**Fig. 8b:**
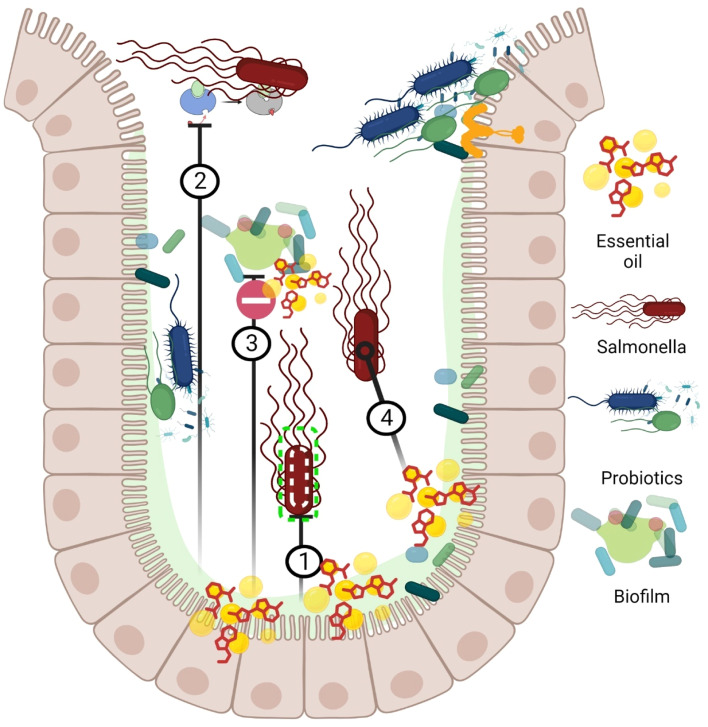
Mechanism of action essential oils / plant extracts approach inhibits *Salmonella* colonization in chicken gastrointestinal tract. ① Interaction with lipid bilayer of *Salmonella* and causing disruption and leakage of cellular components. ② Inhibition of bacterial ATPase enzyme. ③ Disrupt quorum sensing and biofilms. ④ Alteration in bacterial physiology and metabolism by up-regulating stress response genes and downregulating virulence genes.

The efficacy of essential oils in controlling *Salmonella* population in the chicken gut was variable. Hoffman-Pennesi and Wu (2010) reported that the dose of thymol used (400 mg/Kg) may not have been adequate to reduce *Salmonella* population compared with the control. Cerisuelo et al. (2014) reported that increase in the dosage from 50 mg/Kg to 100 mg/kg dose of cinnamaldehyde and thymol performed marginally better in controlling *Salmonella* population. The effect of curcumin on *Salmonella* Enteritidis was variable based on the high standard deviation reported by Hernandez-Patlan et al. (2019). The effectiveness of curcumin on reducing *Salmonella* population in poultry needs more evidence that curcumin could increase pathogenicity and resistance of *Salmonella* by upregulating some genes of antioxidant functions and against antimicrobial peptides, while downregulating genes required for entry and survival in cells (Marathe et al., 2010). Encapsulated essential oil treatments are understood to be more effective as the encapsulation is expected to hold the volatile essential oil in the matrix for a target release at the lower gut (Grilli et al., 2003; Micciche et al., 2019).

### Additive combinations

Several alternative antimicrobials can be incorporated at the pre-harvest stage to mitigate the risk of foodborne pathogen colonization such as *Salmonella* and *Campylobacter* (Van Immerseel et al., 2009; Umaraw et al., 2017). Due to the different mechanisms of action, strategies such as the use of prebiotics, probiotics, organic acids, and essential oils may exert synergistic, additive, or antagonistic effects of the individual products on *Salmonella* colonization in poultry (Vandeplas et al., 2010; Liu et al., 2022). In the literature retrieved for the current systematic review and meta-analysis, essential oils are commonly used in combination with organic acids due to their inherent antimicrobial activity, while prebiotics and prebiotics are used as synbiotics to regulate the intestinal tract homeostasis and improve gut health (Burt, 2004; Micciche et al., 2019; Shah et al., 2023).

**Fig. 9:**
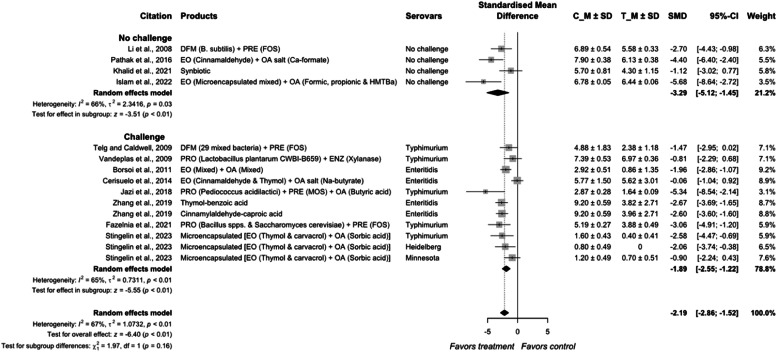
Forest plot of combinations of different additives used to control *Salmonella* colonization in meat-type poultry. Abbreviations: CI, confidence interval; τ^2^, tau-squared; χ^2^, chi-squared; df, degrees of freedom; I^2^, I-squared statistic. The vertical line at the value of zero (0) in the scale of the forest plot is the line of no effect. The hyphen (-) represents a negative standardized mean difference (SMD) effect size and corresponds to a higher *Salmonella* population in control compared with the treatment group. The gray square boxes represent the point estimates of individual studies with a 95 % confidence interval horizontal lines extending on both sides. The mid-point of the gray boxes is the mean effect estimate and the area of the boxes corresponds to the weight of each study. The diamond at the bottom represents the summary estimate and confidence interval of all studies combined. The points on the vertical angle of the diamond represent the overall effect and the width of the diamond represents the 95 % confidence interval.

The estimated effect size of additives combination was −2.19 (95 % CI = −2.86; −1.52) with an overall heterogeneity of 67 %, indicating a medium difference among individual studies for *Salmonella* reduction (*P* < 0.01; Fig. 9). The challenge type of study had an effect size of −1.89 (95 %; CI = −2.55; −1.22) with a heterogeneity of 73 %. The studies in the non-challenge groups were similar in reducing *Salmonella* population (*P* = 0.26).

In the current meta-analysis, combinations of mixed additives by both researchers and manufacturers were evaluated. A total of 12 publications reported combination of additives with organic acids being the most evaluated additive with 8 studies. Nine studies reported both individual and combination effect of additives, with 6 of them reporting a synergistic effect in reducing *Salmonella* colonization. Telg and Caldwell (2009) reported that supplementation of probiotics reduced *Salmonella* colonization, but FOS did not show any effect. No additive effect was observed in feeding birds FOS and probiotics in combination. Similarly, Pathak et al. (2017) reported no synergistic effect from a combination of EO and calcium formate, moreover, the beneficial effect in reducing *Salmonella* colonization was reported mainly from the formic acid supplementation. Cerisuelo et al. (2014) noted that inclusion at 50 mg/kg of EO with sodium butyrate produced a positive effect, whereas the higher dose of 100 mg/kg EO with sodium butyrate or EO alone did not reduce *Salmonella* population, which could be due to its detrimental effect on feed intake or *Salmonella* competing gut microbiota (Abdel-Wareth et al., 2012). However, recent studies have shown the synergistic effect from the combination of additives (Jazi et al., 2018; Fazelnia et al., 2021; Khalid et al., 2021; and Islam et al., 2022). The improved effect may be due to the advantage encapsulation provides in protecting the organic acid and its release in the target section of the intestinal tract, the ceca where the main site of fermentation in poultry gut. (der Poel et al., 2020). Organic acids and essential oils could also disturb ATP metabolism, protein synthesis, and quorum sensing in bacteria (Elshafie and Camele, 2017; Stingelin et al., 2023). An encapsulated product displayed a positive effect in controlling the *Salmonella* population in its preferred persistence and proliferation site, the ceca and such delivery techniques show potential to target pathogens in the lower gut (Stingelin et al. (2023).

Organic acids could alter the *hilA* gene (Van Immerseel et al., 2004) and its combination with essential oil could make environmental conditions such as osmolarity less favorable for *Salmonella* survival and colonization (Borsoi et al., 2011). Essential oils such as carvacrol and thymol are hydrophobic and could integrate into the bacterial cell wall (Lambert et al., 2001), leading to weak integrity of the bacterial cell wall and its lysis (Chauhan and Kang, 2014; Miladi et al., 2016). However, those benefits are not always reflected in the *Salmonella* colonization in the gastrointestinal tract. One common theory reported in those studies is the growth inhibition of detrimental bacteria through the acidification of the lumen content directly through supplementation of organic acids or the metabolites (lactic acid or butyric acid) from beneficial bacteria (Van Immerseel et al., 2006; Knarreborg et al., 2008). Our current findings in the meta-analysis are also in agreement with this observation, with organic acids and probiotics being two of the most effective individual interventions at pre-harvest. Moreover, efficacy of probiotics is improved in combination with prebiotics to form synbiotic (Li et al., 2008; Jazi et al., 2018; Fazelnia et al., 2021; Khalid et al., 2021; and Islam et al., 2022). However, limited information is available on the inclusion rates when cocktails of additives were evaluated. Therefore, future research on the dose effect is necessary for a cost-effective and safety intervention.

### Bacteriophages

**Fig. 10:**
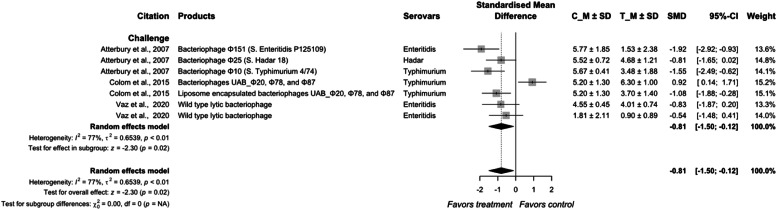
Forest plot of bacteriophage additives used to control *Salmonella* colonization in meat-type poultry. Abbreviations: CI, confidence interval; τ^2^, tau-squared; χ^2^, chi-squared; df, degrees of freedom; I^2^, I-squared statistic. The vertical line at the value of zero (0) in the scale of the forest plot is the line of no effect. The hyphen (-) represents a negative standardized mean difference (SMD) effect size and corresponds to a higher *Salmonella* population in the control compared with the treatment group. The gray square boxes represent the point estimates of individual studies with a 95 % confidence interval horizontal lines extending on both sides. The mid-point of the gray boxes is the mean effect estimate and the area of the boxes corresponds to the weight of each study. The diamond at the bottom represents the summary estimate and confidence interval of all studies combined. The points on the vertical angle of the diamond represent the overall effect and the width of the diamond represents the 95 % confidence interval.

Bacteriophages are host-specific viruses that act as natural parasites of bacteria, they adsorb onto bacterial cell walls, inject their DNA, and utilize the bacterial cell machinery to replicate during the lytic infection cycle and ultimately cause bacteriolysis (Atterbury et al., 2007; Nabil et al., 2018). Since the infection of bacteriophages is host-specific, all the research identified in this systematic review and meta-analysis were challenge studies with *S.* Enteritidis, *S.* Hadar, or *S*. Typhimurium (Fig. 10). The overall effect size of bacteriophages on *Salmonella* reduction in meat-type poultry was −0.81 (95 % CI = −1.50; −0.12) with a heterogeneity of 77 % between the individual studies (*P* < 0.01). Among all studies, Colom et al. (2015) showed a higher population of *Salmonella* in the bacteriophage-treated group. They reported a reduction in *Salmonella* population at 10 d post-challenge, but an increase in the population by 15 d post-challenge compared to control. It is obvious from this observation that the protective action of bacteriophages in the non-encapsulated treatment diminished relatively rapidly. A limitation of the phage therapy is the narrow host range, and the phage can lyse only their homologous hosts, as well as requiring adsorption to the bacterial cell, which can be impeded in the poultry gastric digesta matrix (Greer, 2005).

### Vaccines

**Fig. 11:**
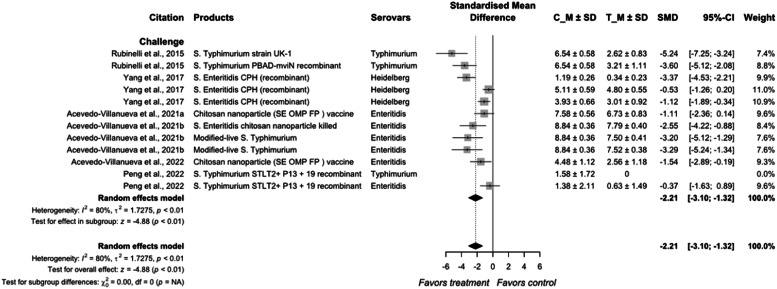
Forest plot of vaccine preparations used to control *Salmonella* colonization in meat-type poultry. Abbreviations: CI, confidence interval; τ^2^, tau-squared; χ^2^, chi-squared; df, degrees of freedom; I^2^, I-squared statistic. The vertical line at the value of zero (0) in the scale of the forest plot is the line of no effect. The hyphen (-) represents a negative standardized mean difference (SMD) effect size and corresponds to a higher *Salmonella* population in the control compared with the treatment group. The gray square boxes represent the point estimates of individual studies with a 95 % confidence interval horizontal lines extending on both sides. The mid-point of the gray boxes is the mean effect estimate and the area of the boxes corresponds to the weight of each study. The diamond at the bottom represents the summary estimate and confidence interval of all studies combined. The points on the vertical angle of the diamond represent the overall effect and the width of the diamond represents the 95 % confidence interval.

Vaccines are biological preparations that resemble the disease-causing microorganism and can contain the microorganism in either live attenuated, inactivated (killed), or recombinant form carrying their subunit proteins or toxins. These are introduced to the host through oral, nasal, or injection (intramuscular, intraperitoneal, and subcutaneous), and could stimulate host immunity to recognize such pathogen and neutralize them when encountered in the future (Marouf et al., 2022). The primary advantage of live-attenuated vaccines is that the vaccinated *Salmonella* strain can replicate, colonize, and infect intestinal and visceral organs (Barrow et al., 1990; Dórea et al., 2010), thereby producing long-lasting protective immunity (Curtiss et al., 1993). These modified live *Salmonella* vaccine strains are not foodborne pathogens, making them a valuable tool for controlling wild-type *Salmonella* at pre-harvest. Vaccine strains isolated from raw poultry products can be identified using whole genome sequencing, allowing distinction between the vaccine strain and the wild type to be considered within the performance standards for poultry processing (USDA FSIS, 2024). However, those vaccines reported from current meta-analysis are not available to the poultry industry yet. Currently, two commercially available killed vaccines, POULVAC® SE and POULVAC® SE-ND-IB, are available for use in broilers and/or layers via intramuscular injection. Live *Salmonella* vaccines, POULVAC® ST and SALMOVAC® SE are available for administration through spray or orally for broilers and layers (Acevedo-Villanueva et al., 2021a). None of the no-challenge studies met the inclusion criteria for the meta-analysis (Fig. 11). In the overall models of the vaccine on *Salmonella* reduction in meat-type poultry, the size of the effect was −2.21 (95 % CI = −3.02; −1.32), with a high heterogeneity of 80 % between the individual studies (*P* < 0.01).

Previous studies on vaccination have been focused on the breeder flocks (Young et al., 2007; Berghaus et al., 2011). Same *Salmonella* serovars found in broiler processing plants can frequently be traced back to both the hatcheries and the parent broiler breeder flocks (Bailey et al., 2002). Vaccination potentially has a central role in reducing *Salmonella* colonization at pre-harvest, because vaccination can reduce both horizontal and vertical transmission of *Salmonella*, particularly for breeder flocks (Inoue et al., 2008; Dórea et al., 2010). With the new framework published by USDA FSIS, *Salmonella* vaccination for broilers has drawn more attention. Additionally, in the latest Constituent Update from USDA FSIS, vaccine strains isolated from raw poultry products will be excluded from the calculation used to categorize establishments under the raw poultry *Salmonella* performance standards from April 2024 (USDA-FSIS, 2024). This regulatory allowance also may encourage the use of vaccination at the broiler level.

Because poultry can harbor a range of *Salmonella* serovars of public health concern, a multivalent vaccine would be required to ensure protection against such a broad range of bacteria (Desin et al., 2013). The efficacy of the vaccines against *Salmonella* serovars in the studies of Yang et al. (2017) and Peng et al. (2022) was numerically noticeable, but not significant. The magnitude of the efficacy of the vaccine would depend on several factors, including the underlying stress and immune status of the birds, and the cross-serovar protection ability of the product against different *Salmonella* serovars based on the homology of antigens in the infecting serovar (Yang et al., 2017; Sharif and Ahmad, 2018; Crouch et al., 2020). Nanoparticles, and phospholipid-calcium cochleate are some of the useful delivery strategies studied for *Salmonella* vaccine in poultry (Acevedo-Villanueva et al., 2021b). While these vaccines have proven effective in controlling *Salmonella* at pre-harvest, further research is needed to determine their efficacy and safety before they can be approved as licensed products.

This meta-analysis provides a better comparison of the ability of different feed additives and strategies to control *Salmonella* population in meat-type birds during the grow-out period. However, inherent variability between the types of birds, the age of birds, duration of study, rearing conditions of the birds, *Salmonella* serovars used and their challenge dose, and the inclusion rates of products used in the studies were included in the meta-analysis (Table S1). A limitation of the publication bias due to the positive results of small studies, and the statistical tests such as funnel plots would only provide a misleading conclusion based on few studies (*n* < 10) and significant heterogeneity (I^2^ > 50 %), thus was not conducted.

## Conclusions

The need to utilize ‘natural’ products and additives as alternatives to antibiotics to address *Salmonella* colonization in poultry is to maintain optimum health status of birds and assure microbiological safety of poultry products. It is evident that the combination of interventions or strategies has shown the highest effect size (SMD = −2.34) for reduction of *Salmonella* population, followed by the vaccines (SMD = −2.21) and the organic acids (SMD = −2.11) in meat-type poultry. The use of probiotics, prebiotics and essential oils has a marked effect in the reduction of *Salmonella* in poultry; however, their combination has a more pronounced impact.

It is evident from the forest plots that variability in the response exists for all the additives. The intention of this systematic review and meta-analysis is to provide a perspective for exploring interventions to reduce the prevalence and/or concentrations of *Salmonella* at pre-harvest. Future research should consider standardizing the minimum replicate size and removing any confounding factors to ensure inclusion in the meta-analysis and for robust comparison. More rigorous evaluations should be included in the research studies to properly define the minimum effective dosage of the individual additive/product prior to recommending their extensive use in poultry production. The use of AGP alternatives has provided the poultry industry resources to address the challenge of *Salmonella* colonization and exploit multi-hurdle intervention strategy to reduce its load ante-mortem to allow further reductions during poultry processing to enhance the microbiological safety of poultry products.

## CRediT authorship contribution statement

**Amit K. Singh:** Conceptualization, Investigation, Methodology, Validation, Writing – original draft. **Jinquan Wang:** Conceptualization, Formal analysis, Investigation, Methodology, Software, Validation, Visualization, Writing – review & editing. **Pranita S. Patil:** Investigation. **Deepak Subedi:** Investigation. **Bharath Mallavarapu:** Investigation. **Sujitha Bhumanapalli:** Investigation. **Sasikala Vaddu:** Investigation. **Rami A. Dalloul:** Validation, Writing – review & editing. **Manpreet Singh:** Project administration, Supervision, Writing – review & editing. **Harshavardhan Thippareddi:** Conceptualization, Investigation, Methodology, Project administration, Resources, Supervision, Writing – review & editing.

## Disclosures

This letter is to inform you that we are respectfully submitting our manuscript titled “A systematic review and meta-analysis of the efficacy of alternatives to antibiotic growth promoters as strategies to reduce Salmonella in meat-type poultry (pre-harvest)” for review.

## Appendix Supplementary materials

Image, application 1

## Footnotes

Supplementary material associated with this article can be found, in the online version, at doi:10.1016/j.psj.2025.105640.
