# Supplementary material for: A systematic review and meta-analysis of the efficacy of alternatives to antibiotic growth promoters as strategies to reduce Salmonella in meat-type poultry (pre-harvest)
Source: Poult Sci. 2025 Aug 6;104(11):105640. doi: 10.1016/j.psj.2025.105640 (PMC12455111; doi:10.1016/j.psj.2025.105640)
Supplement: Supplementary file 1 [file mmc1.docx]

**Table S1.** Meta-analysis table of detailing product types, doses, rearing conditions of birds, sampling age and type, and replicates used in the experiments in meat-type poultry challenged or not with Salmonella serovars.

| Products | Product dose | Serovar | Challenge day and dose | Rearing condition and bird type | Starting age, sampling day, and type | Replicate (n & m) | References |
| --- | --- | --- | --- | --- | --- | --- | --- |
| Combinations |  |  |  |  |  |  |  |
| EO (Cinnamaldehyde & Thymol) + OA salt (Na-butyrate) | 50 mg/kg EO and 1g/kg Na-butyrate (EOB50) in feed | Enteritidis | 7 d; 2.83 × 10^8 CFU/mL orally | Pens; Hubbard male broilers | 1 d; 37 d; Feces | n = 8, m = 12 | Cerisuelo et al., 2014 |
| EO (Cinnamaldehyde & Thymol) + OA salt (Na-butyrate) | 100 mg/kg EO and 1g/kg Na-butyrate (EOB50) in feed | Enteritidis | 7 d; 2.83 × 10^8 CFU/mL orally | Pens; Hubbard male broilers | 1 d; 37 d; Feces | n = 8, m = 12 | Cerisuelo et al., 2014 |
| EO (Cinnamaldehyde) + OA salt (Ca-formate) | NC + a combination of cinnamaldehyde (15%) and calcium formate, (20%); Amaril, Dex Iberica, SA Tarragona, Spain; 500 mg/kg feed | No challenge | N/A | Pens; Cobb 400 mixed-sex broilers | 1 d; 40 d; Ceca | n = 8, m = 10 | Pathak et al., 2016 |
| EO (Microencapsulated mixed) + OA (Formic, propionic & HMTBa) | Organic acid (OA): 200 mg/kg + Essential oil (EO): 150 mg/kg feed | No challenge | N/A | Pens, open-sided house; Cobb 500 broilers | 1 d; 35 d; Ileal digesta | n = 6, m = 20 | Islam et al., 2022 |
| EO (Mixed) + OA (Mixed) | OA- 0.5 g/kg (0.05%) and EO- 1 g/kg (0.1%) in feed | Enteritidis | 1 d; 1 × 10^5 CFU/mL orally | Cages; Ross broilers | 1 d; 21 d; Cloacal swab | n = 15, m = 1 | Borsoi et al., 2011 |
| EO (Mixed) + OA (Mixed) | OA and EA 1 g/kg (0.1%) in feed | Enteritidis | 1 d; 1 × 10^5 CFU/mL orally | Cages; Ross broilers | 1 d; 21 d; Cloacal swab | n = 15, m = 1 | Borsoi et al., 2011 |
| Microencapsulated [EO (Thymol & carvacrol) + OA (Sorbic acid)] | 2 g/kg (0.2%) from 1-21 d and 1 g/kg (0.1%) from 35 -42 d in feed microencapsulated (sorbic acid (25%), thymol (9.5%) and carvacrol (2.5%)) | Typhimurium | 3 and 33 d; 0.5 mL of 1 × 10^8 CFU/bird orally | NA; Commercial broilers | 1 d; 42 d; Ceca | n = m = 5 | Stingelin et al., 2023 |
| Microencapsulated [EO (Thymol & carvacrol) + OA (Sorbic acid)] | 2 g/kg (0.2%) from 1-21 d and 1 g/kg (0.1%) from 35 -42 d in feed microencapsulated (sorbic acid (25%), thymol (9.5%) and carvacrol (2.5%)) | Heidelberg | 3 and 33 d; 0.5 mL of 1 × 10^8 CFU/bird orally | NA; Commercial broilers | 1 d; 42 d; Ceca | n = m = 5 | Stingelin et al., 2023 |
| Microencapsulated [EO (Thymol & carvacrol) + OA (Sorbic acid)] | 2 g/kg (0.2%) from 1-21 d and 1 g/kg (0.1%) from 35 -42 d in feed microencapsulated (sorbic acid (25%), thymol (9.5%) and carvacrol (2.5%)) | Minnesota | 3 and 33 d; 0.5 mL of 1 × 10^8 CFU/bird orally | NA; Commercial broilers | 1 d; 42 d; Ceca | n = m = 5 | Stingelin et al., 2023 |
| PRO *(Bacillus* *spp.* & *S*. *cerevisiae* + PRE (FOS) | 0.2 g/kg (0.02%) feed | Typhimurium | 21 d; 0.5 mL 1 × 10^6 CFU/mL/bird orally | Cages; Ross 308 broilers | 1 d; 26 d; Ceca | n = 6, m = 10 | Fazelnia et al., 2021 |
| PRO (*Lactobacillus* *plantarum* CWBI-B659) + ENZ (Xylanase) | L. plantarum 1 × 10^9 CFU/kg feed + xylanase 105000 IU per kg feed (0.01%) | Typhimurium | 3 d; 1 × 10^8 CFU/bird orally | Cages; Ross male broilers | 1 d; 30 d; Ceca | n = 4, m = 6 | Vandeplas et al., 2009 |
| PRO (*Pediococcus* *acidilactici*) + PRE (MOS) + OA (Butyric acid) | *Pediococcus acidilactici* 1 g/kg (0.01%; 1× 10^10 CFU/g), MOS 2 g/kg (0.2%) and encapsulated butyric acid (50% butyrate salt) 5 g/kg (0.05%) in feed | Typhimurium | 3 d; 0.5 mL of 1 × 10^5 CFU/mL/bird orally | Pens; Ross 308 broilers | 1 d; 21 d; Ceca | n = 5, m = 14 | Jazi et al., 2018 |
| DFM (29 mixed bacteria) + PRE (FOS) | 1 manufacturer dose of probiotics and FOS 9.9 g/kg (0.99%) in feed | Typhimurium | 1 d; 0.5 mL of 10^4 CFU/bird orally | Pens; Cobb broilers | 1 d; 8 d; Ceca | n = m = 5 | Telg and Caldwell, 2009 |
| DFM (*B*. *subtilis*) + PRE (FOS) | *Bacillus subtilis* 1 g/kg (0.1%) + FOS 3 g/kg (0.3%) in feed | No challenge | N/A | Cages; Arbor Acres broilers | 1 d; 42 d; Ceca | n = 3 (pooled) | Li et al., 2008 |
| Probiotics |  |  |  |  |  |  |  |
| Mix probiotics (*B*. *subtilis*, *Clostridium* *butyricum* & *Enterococcus* *faecalis*) | 0.5 g/kg (0.05%) of *Bacillus subtilis* (2 × 10^8 CFU/g), *Clostridium butyricum* (2 × 10^6 CFU/g), and *Enterococcus faecalis* (1 × 10^6 CFU/g) in feed | No challenge | N/A | Pens; AA+ broilers | 1 d; 42 d; Feces | n = 6, m = 20 | Zou et al., 2022a |
| Mix probiotics (*B*. *subtilis*, *C*. *butyricum* & *E*. *faecalis*) | 2 g/kg (0.2%) of *Bacillus subtilis* (2 × 10^8 CFU/g), *Clostridium butyricum* (2 × 10^6 CFU/g), and *Enterococcus faecalis* (1 × 10^6 CFU/g) in feed | No challenge | N/A | Pens; AA+ broilers | 1 d; 42 d; feces | n = 6, m = 20 | Zou et al., 2022b |
| Mix probiotics (*Bacillus* *spp.* & *S*. *cerevisiae*) | 0.2 g/kg feed of probiotic blend containing *Bacillus subtilis* 4×10^8 CFU/g, *Bacillus licheniformis* 3×10^9 CFU/g, and *Saccharomyces cerevisiae* 1×10^8 CFU/g | Typhimurium | 21 d; 0.5 mL 1 × 10^6 CFU/mL/bird orally | Cages; Ross 308 broilers | 1 d; 26 d; Ceca | n = 6, m = 10 | Fazelnia et al., 2021 |
| *B*. *subtilis mix* 3 strains (NP122, B2 & AM0904) | 0.5 g/kg feed of probiotic blend containing *Bacillus subtilis* 3 strains (NP122, B2, and AM0904) supplying 2.0 × 10^6 CFU/kg feed | Heidelberg | 3 d; 1 mL of 1 × 10^7 CFU/g/bird orally | Cages; Cobb 500 male broilers | 1 d; 21 d; Ceca | n = 4, m = 12 | Hayashi et al., 2018 |
| Mix probiotics (*L*. *acidophilus*, *B*. *subtilis* & *A*. *niger*) | 340 g/kg (34%) fermented rapeseed meal (FRSM) in feed with 10^5 CFU mixed culture of *Lactobacillus acidophilus* (PTCC1643), *Bacillus subtilis* (PTCC1156), and *Aspergillus niger* (PTCC5010) per kg FRSM | Typhimurium | 3 d; 0.5 mL of 1 × 10^5 CFU/bird orally | Pens; Cobb 500 male broilers | 1 d; 10 d; Ceca | n = 4, m = 10 | Ashayerizadeh et al., 2017 |
| Mix LAB probiotics | 0.2 g/kg (0.02%) feed of lactic acid bacteria (LAB) containing 5.2 × 10^10 CFU/g of *Lactobacillus acidophilus*, *Lactobacillus casei*, *Bifidobacterium thermophilum*, and *Enterococcus faecium* | Typhimurium | 3 d; 0.5 mL 1 × 10^6 CFU/mL/bird orally | Cages; Ross 308 broilers | 1 d; 24 d; Ceca | n = 3, m = 15 | Jazi et al., 2019 |
| *B*. *lichenifornis* & *B*. *subtilis* | *Bacillus licheniformis* 6.6×10^5 and *Bacillus subtilis* 3.3×10^5 CFU/kg feed | No challenge | N/A | N/A; Chinese Huainan Partridge Shank chickens | 1 d; 56 d; Ceca | n = 5, m = 20 | Yang et al., 2017 |
| *B*. *subtilis* & *B*. *licheniformis* | *Bacillus subtilis* @ 1.4 × 10^9 CFU/kg feed, and *Bacillus licheniformis* 8.2 × 10^8 CFU/kg feed | No challenge | N/A | Pens; Ross 308 male broilers | 1 d; 35 d; Ceca | n = 10, m = 18 | Biswas et al., 2022 |
| *B*. *subtilis* B2A | *Bacillus subtilis* B2A @ 1 × 10^5 CFU/g of feed | No challenge | N/A | Cages; Ross 308 male broilers | 1 d; 28 d; Large intestine | n = 9, m = 16 | Park et al., 2014 |
| *B*. *subtilis* RX7 | 1 g/kg (0.1%) feed of probiotic blend containing *Bacillus subtilis* RX7 1.0 × 10^9 CFU/g | No challenge | N/A | Cages; Ross 308 broilers | 1 d; 35 d; Ceca | n = 6, m = 12 | Park et al., 2018 |
| *B*. *subtilis* C14 | 1 g/kg (0.1%) feed of probiotic blend containing *Bacillus subtilis* C14 1.0 × 10^9 CFU/g | No challenge | N/A | Cages; Ross 308 broilers | 1 d; 35 d; Ceca | n = 6, m = 12 | Park et al., 2018 |
| *B*. *subtilis* KT260179 | 340 g/kg (0.34%) of 2× 10^9 CFU/g *Bacillus subtilis* KT260179 in feed | No challenge | N/A | N/A; Chinese Huainan Partridge chickens | 1 d; 56 d; Ceca | n = 6, m = 25 | Yang et al., 2016 |
| *B*. *subtilis* | 0.25 g/kg (0.025%) of *Bacillus subtilis* preparation containing 2.0 × 10^10 CFU/g in feed | No challenge | N/A | Cages; Arbor Acres (AA) broilers | 1 d; 42 d; Ceca | n = 4, m = 40 | Gao et al., 2017 |
| *B*. *subtilis* | 0.2 g/kg (0.02%) of *Bacillus subtilis* preparation containing 2.0 × 10^10 CFU/g in feed | No challenge | N/A | Cages; Arbor Acres (AA) broilers | 1 d; 42 d; Ceca | n = 4, m = 40 | Gao et al., 2017 |
| *B*. *subtilis* | 1 g/kg (0.1%) of *Bacillus subtilis* containing 6 × 10^10 CFU/g in feed | No challenge | N/A | Cages; Arbor Acres broilers | 1 d; 42 d; Ceca | n = 3 (pooled) | Li et al., 2008 |
| *L*. *reuteri* | 1 g/kg (0.1%) commercial DFM containing *Lactobacillus reuteri* in feed | No challenge | N/A | Pens; Ross 308 broilers | 1 d; 35 d; Ceca | n = 4, m = 50 | Salim et al., 2013 |
| *Lactobacillus* *reuteri* *S5* | *Lactobacillus reuteri* S5 @ 1.5 × 10^8 CFU/mL oral/day for 14 d | Enteritidis | 1 – 14 d; 1 × 10^9 CFU/mL orally | Cages; Broilers | 1 d; 14 d; Ceca | n = 9, m = 5 | Shi et al., 2022 |
| *L*. *plantarum* CWBI-B659 (P) | *Lactobacillus* *plantarum* CWBI-B659 (P) @ 1 × 10^9 CFU/kg feed | Typhimurium | 3 d; 1 × 10^8 CFU/bird orally | Cages; Ross male broilers | 1 d; 30 d; Ceca | n = 4, m = 6 | Vandeplas et al., 2009 |
| *L*. *Casei* ATCC 334 | *Lactobacillus casei* ATCC 334 @ 0.1 mL of 1× 10^10 CFU/mL/bird for d 1-7 in feed | No challenge | N/A | Cages; SPF chicks | 1 d; 28 d; Ceca | n = 8, m = 1 | Tabashsum et al., 2020 |
| *L*. *Casei* +mcra | *Lactobacillus casei* +mcra @ 0.1 mL of 1× 10^10 CFU/mL/bird for d 1-7 in feed | No challenge | N/A | Cages; SPF chicks | 1 d; 28 d; Ceca | n = 8, m = 1 | Tabashsum et al., 2020 |
| *Pediococcus* *acidilactici* | 0.1 g/kg (0.01%) feed of probiotic blend containing *Pediococcus acidilactici* | Typhimurium | 3 d; 0.5 mL of 1 × 10^5 CFU/mL/bird orally | Pens; Ross 308 broilers | 1 d; 21 d; Ceca | n = 5, m = 14 | Jazi et al., 2018 |
| *Escherichia* *coli* Nissle 1917 (EcN) | *Escherichia coli* strain, Nissle 1917 (EcN) 1 mL of 1 × 10^7 CFU/bird on d 1 orally | Enteritidis | 1 d; 1 mL of 1 × 10^7 CFU/bird orally | Cages; Turkey | 4 d; 14 d; Ceca | n = 5, m = 1 | Forkus et al., 2017 |
| *E*. *coli* Nissle 1917 with Microcin J25 (EcNJ25) | *Escherichia coli* strain, Nissle 1917 with Microcin J25 (EcNJ25) 1 mL of 1 × 10^7 CFU/bird on d 1 orally | Enteritidis | 1 d; 1 mL of 1 × 10^7 CFU/bird orally | Cages; Turkey | 4 d; 14 d; Ceca | n = 5, m = 1 | Forkus et al., 2017 |
| *C*. *butyricum* | *Clostridium butyricum* @ 1 × 10^7 CFU/kg feed | No challenge | N/A | Cages; Lingnan Yellow male broilers | 1 d; 42 d; Ceca | n = 6, m = 15 | Yang et al., 2012 |
| *C*. *butyricum* | *Clostridium butyricum* @ 2 × 10^7 CFU/kg feed | No challenge | N/A | Cages; Lingnan Yellow male broilers | 1 d; 42 d; Ceca | n = 6, m = 15 | Yang et al., 2012 |
| *C. butyricum* | *Clostridium butyricum* @ 3 × 10^7 CFU/kg feed | No challenge | N/A | Cages; Lingnan Yellow male broilers | 1 d; 42 d; Ceca | n = 6, m = 15 | Yang et al., 2012 |
| *E*. *faecium* | *Enterococcus faecium* NCIMB 11181 @ 4 × 10^8 CFU/kg of feed | Typhimurium | 10 and 11 d; 1 mL 1 × 10^9 CFU/mL orally | Cages; Arbor Acor AA+ male broilers | 1 d; 7 d; Ceca | n = 6, m = 10 | Shao et al., 2022 |
| *B*. *velezensis* CE 100 | *Bacillus velezensis* CE 100 @ 1 × 10^5 CFU/mL and product added 10 g/kg (1%) of feed | No challenge | N/A | Pens; Ross 308 broilers | 2 d; 43 d; Ceca | n = 2, m = 11 | Park et al., 2022 |
| *S*. *cerevisiae* | *Saccharomyces cerevisiae* @ 2 × 10^12 CFU/kg feed | No challenge | N/A | Pens; Ross 308 broilers | 1 d; 35 d; Ceca | n = 3, m = 1 | Khalid et al., 2021 |
| Prebiotics |  |  |  |  |  |  |  |
| Fructo-oligosaccharides (FOS) | 9.9 g/kg (0.99%) fructo-oligosaccharides (FOS) in feed | Typhimurium | 1 d; 0.5 mL of 10^4 CFU/bird orally | Pens; Cobb broilers | 1 d; 8 d; Ceca | n = m = 5 | Telg and Caldwell, 2009 |
| Fructo-oligosaccharides (FOS) | 3 g/kg (0.3%) fructo-oligosaccharides (FOS) in feed | No challenge | N/A | Cages; Arbor Acres broilers | 1 d; 42 d; Ceca | n = 3 (pooled) | Li et al., 2008 |
| Mannan oligosaccharides | 2 g/kg (0.2%) active mannan oligosaccharides (MOS) in feed | Typhimurium | 3 d; 0.5 mL of 1 × 10^5 CFU/mL/bird orally | Pens; Ross 308 broilers | 1 d; 21 d; Ceca | n = 5, m = 14 | Jazi et al., 2018 |
| Wheat bran 280 μm particle size | 10 g/kg (1%) of 280 μm size wheat bran in feed | Enteritidis | 10 d; 10^8 CFU per bird orally | Pens; Ross 308 broilers | 1 d; 14 d; Ceca | n = m = 20 | Vermeulen et al., 2017 |
| Wheat bran 280 μm particle size | 10 g/kg (1%) of 280 μm size wheat bran in feed | Enteritidis | 10 d; 10^8 CFU per bird orally, seeder | Pens; Ross 308 broilers | 1 d; 42 d; Ceca | n = m = 20 | Vermeulen et al., 2018 |
| Wheat bran 1690 μm particle size | 10 g/kg (1%) of 1690 μm size wheat bran in feed | Enteritidis | 10 d; 10^8 CFU per bird orally, seeder | Pens; Ross 308 broilers | 1 d; 42 d; Ceca | n = m = 20 | Vermeulen et al., 2019 |
| Trehalose | 50 g/kg (5%) trehalose in feed | Typhimurium | 28 d; 3.5 × 10^8 CFU/mL/bird orally | Pens; Arbor Acres male broilers | 1 d; 35 d; Ceca | n = m = 15 (Control), n = m = 8 (product) | Wu et al., 2020 |
| Gum Arabica | 10 g/kg (1%) Gum Arabica in feed | No challenge | N/A | Cages; Ross 308 male broilers | 1 d; 10d d; Ceca | n = 12, m = 6 | Al-Baadani et al., 2022 |
| Red Seaweed | 2 g/kg (0.2%) red seaweed in feed | No challenge | N/A | Cages; Ross 308 broilers | 1 d; 42 d; Feces | n = 8, m = 18 | Balasubramanian et al., 2021 |
| Asparagus | 50 g/kg (5%) trimmed asparagus byproduct in feed | No challenge | N/A | Cages; Ross 308 broilers | 1 d; 21 d; Ceca | n = 4, m = 20 | Nopparatmaitree et al., 2022 |
| Organic acids |  |  |  |  |  |  |  |
| Mix organic acids (fumaric, citric, malic & MCFAs: capric & caprylic) | 0.5 g/kg (0.05%) coated organic acid with 17% fumaric acid, 13% citric acid, 10% malic acid, and 1.2% MCFAs (capric and caprylic acid, provided as a 1:1 mixture product) in feed | No challenge | N/A | Pens; Ross 308 unsexed broilers | 1 d; 49 d; Excreta | n = 10, m = 16 | Nguyen and Kim, 2020 |
| Mix organic acids (fumaric, citric, malic & MCFAs: capric & caprylic) | 0.75 g/kg (0.075%) coated organic acid with 17% fumaric acid, 13% citric acid, 10% malic acid, and 1.2% MCFAs (capric and caprylic acid, provided as a 1:1 mixture product) in feed | No challenge | N/A | Pens; Ross 308 unsexed broilers | 1 d; 49 d; Excreta | n = 10, m = 16 | Nguyen and Kim, 2020 |
| Mix organic acids (fumaric, citric, malic & MCFAs: capric & caprylic) | 1 g/kg (0.1%) coated organic acid with 17% fumaric acid, 13% citric acid, 10% malic acid, and 1.2% MCFAs (capric and caprylic acid, provided as a 1:1 mixture product) in feed | No challenge | N/A | Pens; Ross 308 unsexed broilers | 1 d; 49 d; Excreta | n = 10, m = 16 | Nguyen and Kim, 2020 |
| Mix organic acids (propionic, formic, and HMTBa) | 0.2 g/kg (0.02%) organic acids mixture of propionic acid, formic acid and HMTBa in feed | No challenge | N/A | Pens, open-sided house; Cobb 500 broilers | 1 d; 35 d; Ileal digesta | n = 6, m = 20 | Islam et al., 2022 |
| Mix organic acids & salt (formic, propionic & Ca propionate) | 20 g/kg (2%) of the organic acid mixture (formic acid 15%+ propionic acid 3% + calcium propionate 3%) in feed | No challenge | N/A | Pens; Mulard ducks | 1 d; 42 d; Ceca | n = 10, m = 10 | Ibrahim et al., 2020 |
| Mix organic acids & salt encapsulated in vegetable oil | 0.6 g/kg (0.06%) of Galliacid^®^ containing organic acids and salts encapsulated in oil in feed | No challenge | N/A | Pens; Ross 308 unsexed broilers | 1 d; 35 d; Ceca | n = 5, m = 20 | Hassan et al., 2010 |
| Mix organic acids, salt & flavoring agent encapsulated in vegetable oil | 1 g/kg (0.1%) of Biacid^®^ containing organic acids and salts encapsulated in oil in feed | No challenge | N/A | Pens; Ross 308 unsexed broilers | 1 d; 35 d; Ceca | n = 5, m = 20 | Hassan et al., 2010 |
| Formic acid | 5 mL/L (0.5%) formic acid in drinker water | Typhimurium | 35 and 41 d; 1 × 10^8 CFU/mL/bird orally | Pens; Broilers | 35 d; 42 d; Ceca | n = 5, m = 10(20) | Byrd et al., 2001 |
| Acetic acid | 5 mL/L (0.5%) acetic acid in drinker water | Typhimurium | 35 and 41 d; 1 × 10^8 CFU/mL/bird orally | Pens; Broilers | 35 d; 42 d; Ceca | n = 5, m = 10(20) | Byrd et al., 2001 |
| Butyric acid | 5 g/kg (0.5%) butyric acid in feed | Typhimurium | 3 d; 0.5 mL of 1 × 10^5 CFU/mL/bird orally | Pens; Ross 308 male broilers | 1 d; 21 d; Ceca | n = 5, m = 14 | Jazi et al., 2018 |
| Lactic acid | 5 mL/L (0.5%) lactic acid in drinker water | Typhimurium | 35 and 41 d; 1 × 10^8 CFU/mL/bird orally | Pens; Broilers | 35 d; 42 d; Ceca | n = 5, m = 10(20) | Byrd et al., 2001 |
| Ascorbic acid | 0.1 g/kg (0.01%) ascorbic acid in feed | Enteritidis | 6 d; 1 × 10^7 CFU/bird orally | Cages; Cobb male broilers | 1 d; 16 (10 dpc) d; Ceca | n = m = 4 | Hernandez-Patlan et al., 2019 |
| Caprylic acid | 10 g/kg (1%) caprylic acid in feed from d 37-42 | Enteritidis | 25 d; 1 × 10^8 CFU/mL/bird orally | Pens; Cornish cross broilers | 1 d; 42 d; Ceca | n = 10 | Kollanoor-Johny et al., 2012 |
| Ca formate | 0.5 g/kg (0.05%) calcium formate-20% in feed | No challenge | N/A | Pens; Cobb 400 mixed-sex broilers | 1 d; 40 d; Ceca | n = 8, m = 10 | Pathak et al., 2016 |
| Essential oils |  |  |  |  |  |  |  |
| Trans-cinnamaldehyde + Eugenol | 10 g/kg (1%) mix of trans-cinnamaldehyde (TC) and eugenol (EG) given from d 37 to 42 in feed | Enteritidis | 30 d; 1 × 10^8 CFU/mL/bird orally | Pens; Ross broilers | 1 d; 42 d; Ceca | n = m = 10 | Kollanoor-Johny et al., 2012 |
| Trans-cinnamaldehyde + Eugenol | 7.5 g/kg (0.75%) mix of trans-cinnamaldehyde (TC) and eugenol (EG) given from d 37 to 42 in feed | Enteritidis | 30 d; 1 × 10^8 CFU/mL/bird orally | Pens; Ross broilers | 1 d; 42 d; Ceca | n = m = 10 | Kollanoor-Johny et al., 2012 |
| Cinnamaldehyde + thymol | 0.05 g/kg (0.005%) cinnamaldehyde and thymol in feed | Enteritidis | 7 d; 2.83 × 10^8 CFU/mL/bird orally | Pens; Hubbard male broilers | 1 d; 37 d; Feces | n = 8, m = 12 | Cerisuelo et al., 2014 |
| Cinnamaldehyde + thymol | 0.1 g/kg (0.01%) cinnamaldehyde and thymol in feed | Enteritidis | 7 d; 2.83 × 10^8 CFU/mL/bird orally | Pens; Hubbard male broilers | 1 d; 37 d; Feces | n = 8, m = 12 | Cerisuelo et al., 2014 |
| Thymol | 0.4 g/kg (0.04%) thymol in feed | Enteritidis | 3 d; 1 × 10^4 CFU/mL/bird orally | Pens; Mixed sex SPF chicks | 1 d; 14 d; Ceca | n = m = 10 | Hoffman-Pennesi and Wu, 2010 |
| Thymol | 0.8 g/kg (0.08%) thymol in feed | Enteritidis | 3 d; 1 × 10^4 CFU/mL/bird orally | Pens; Mixed sex SPF chicks | 1 d; 14 d; Ceca | n = m = 10 | Hoffman-Pennesi and Wu, 2010 |
| Carvacrol, thymol, and paracymene mix | 300μL carvacrol, thymol, and paracymene mix essential oils daily orally for 14 d | No challenge | N/A | Cages; Ross 308 mixed sex broilers | 28 d; 42 d; Intestinal digesta | n = 10, m = 1 | Liu et al., 2018 |
| Carvacrol, thymol, and paracymene mix | 400μL carvacrol, thymol, and paracymene mix essential oils daily orally for 14 d | No challenge | N/A | Cages; Ross 308 mixed sex broilers | 28 d; 42 d; Intestinal digesta | n = 10, m = 1 | Liu et al., 2018 |
| Microencapsulated thymol | 1 mL/L (0.1%) microencapsulated thymol oil in drinking water from 5 dpc for 3 d | Enteritidis | 15 d; 1 × 10^9 CFU/mL/bird orally | Pens; Cobb broilers | 1 d; 42 (28 dpc) d; Ceca | n = m = 5 | Hamed et al., 2022 |
| Microencapsulated oil mix from oregano, rosemary, cinnamon, and chili pepper | 0.15 g/kg (0.015%) microencapsulated photo essential oil mix from oregano, rosemary, cinnamon, and chili pepper extract in feed | No challenge | N/A | Pens, open-sided house; Cobb 500 broilers | 1 d; 35 d; Ileal digesta | n = 6, m = 20 | Islam et al., 2022 |
| Microencapsulated oil mix from thyme, savory, peppermint, and black pepper | 1 g/kg (0.1%) microencapsulated essential oil mix from thyme, savory, peppermint, and black pepper in feed | Enteritidis | 3 d; 1 × 10^9 CFU/mL/bird orally | Pens; Ross 308 male broilers | 1 d; 10 d; Ceca | n = 5, m = 10 | Moharreri et al., 2022 |
| Microencapsulated oil mix from thyme, savory, peppermint, and black pepper | 2 g/kg (0.2%) microencapsulated essential oil mix from thyme, savory, peppermint, and black pepper in feed | Enteritidis | 3 d; 1 × 10^9 CFU/mL/bird orally | Pens; Ross 308 male broilers | 1 d; 10 d; Ceca | n = 5, m = 10 | Moharreri et al., 2022 |
| Coriander oil | 25 μL/L (0.0025%) Coriander essential oil nanoemulsion in drinking water | No challenge | N/A | Pens; New Lohman male broilers | 1 d; 35 d; Jejunum | n = 4, m = 9 | Sholiha et al., 2023 |
| Curcumin | 0.1 g/kg (0.01%) curcumin in feed | Enteritidis | 6 d; 1 × 10^7 CFU per bird orally | Cages; Cobb male broilers | 1 d; 16 (10 dpc) d; Ceca | n = m = 9 | Hernandez-Patlan et al., 2019 |
| Bacteriophages |  |  |  |  |  |  |  |
| Bacteriophage Φ151 (*S. enterica* Enteritidis P125109) | 1 mL PBS containing 30% (wt/vol) CaCO3 containing 1 × 10^11 PFU/mL/bird on d 38 orally | Enteritidis | 36 d; 1 × 10^8 CFU/mL/bird orally | Pens; Ross broiler | 34 d; 2 d; Ceca | n = 12, m = 3 | Atterbury et al., 2007 |
| Bacteriophage Φ10 (*S. enterica* Typhimurium 4/74) | 1 mL PBS containing 30% (wt/vol) CaCO3 containing 1 × 10^11 PFU/mL/bird d 38 orally | Typhimurium | 36 d; 1 × 10^8 CFU/mL/bird orally | Pens; Ross broiler | 34 d; 2 d; Ceca | n = 12, m = 3 | Atterbury et al., 2007 |
| Wild-type lytic bacteriophage | 2.9 × 10^10 PFU/bird for 5 d from d 6 – 10 orally | Enteritidis | 1 d; 6 × 10^6 CFU/mL/bird orally | Cages; Embrapa 021 broilers | 1 d; 20 d (10 dpc) d; Ceca | n = 8, m = 1 | Vaz et al., 2020 |
| Wild-type lytic bacteriophage | 6.8 × 10^10 PFU/ bird for 5 d from d 31 – 35 orally | Enteritidis | 1 d; 6 × 10^6 CFU/mL/bird orally | Cages; Embrapa 021 broilers | 1 d; 45 d (10 dpc) d; Ceca | n = 9, m = 1 | Vaz et al., 2020 |
| Bacteriophages UAB_Φ20, Φ78, and Φ87 | 0.1 mL of 10^11 PFU/mL/bird from d 1 – 7 orally | Typhimurium | 1 d; 0.1 mL of 1×10^8 CFU/mL/bird orally | Pens; Ross 308 broilers | 1 d; 12 d (10 dpc) d; Ceca | n = m = 14 | Colom et al., 2015 |
| Bacteriophages UAB_Φ20, Φ78, and Φ87 | 0.1 mL of 10^11 PFU/mL/bird from d 1 – 7 orally | Typhimurium | 1 d; 0.1 mL of 1×10^8 CFU/mL/bird orally | Pens; Ross 308 broilers | 1 d; 17 d (15 dpc) d; Ceca | n = m = 14 | Colom et al., 2015 |
| Liposome encapsulated bacteriophages UAB_Φ20, Φ78, and Φ87 | 0.1 mL of 10^11 PFU/mL/bird from d 1 – 7 orally | Typhimurium | 1 d; 0.1 mL of 1×10^8 CFU/mL/bird orally | Pens; Ross 308 broilers | 1 d; 12 d (10 dpc) d; Ceca | n = m = 14 | Colom et al., 2015 |
| Liposome encapsulated bacteriophages UAB_Φ20, Φ78, and Φ87 | 0.1 mL of 10^11 PFU/mL/bird from d 1 – 7 orally | Typhimurium | 1 d; 0.1 mL of 1×10^8 CFU/mL/bird orally | Pens; Ross 308 broilers | 1 d; 17 d (15 dpc) d; Ceca | n = m = 14 | Colom et al., 2015 |
| Bacteriophage P1:1 | 10^10 PFU/kg feed | Enteritidis | 1 d; 10^4 CFU/bird orally | Cages; Broilers | 1 d; 14 d; Ceca | n = m = 10 | Sklar and Joerger, 2001 |
| Bacteriophage (EP2+MUT3+M4+YP) | 2 × 10^12/kg feed | Enteritidis | 1 d; 10^8 CFU/bird orally | Cages; Broilers | 1 d; 14 d; Ceca | n = m = 10 | Sklar and Joerger, 2001 |
| Vaccines |  |  |  |  |  |  |  |
| *Salmonella* Enteritidis CPH (recombinant) | 1.6 × 10^7 CFU/bird (estimated) on d 1 as spray | Heidelberg | 14 d; 3 × 10^7 CFU/bird orally | Pens; Broilers | 1 d; 25 d (12 dpc) d; Ceca | n = m = 15 | Yang et al., 2017 |
| Chitosan nanoparticle (SE OMP FP) vaccine | 0.02 mg/bird booster on d 1 & 7, sprayed on d 1 and orally on d7 | Enteritidis | 14 d; 1 × 10^7 CFU/bird orally | Pens; Cobb broilers | 1 d; 35 (21 dpc) d; Ceca | n = 6, m = 16 | Acevedo-Villanueva et al., 2022 |
| Chitosan nanoparticle (SE OMP FP) vaccine | 1 mg (0.2 mL) in amniotic cavity on ED 18 | Enteritidis | 7 d; 1 × 10^9 CFU/bird orally | Pens; Cobb broilers | ED 18, d 1 d; 21 (14 dpc) d; Ceca | n = 6, m = 11 | Acevedo-Villanueva et al., 2021 |
| *Salmonella* Enteritidis chitosan nanoparticle killed | 0.01 mg/mL/bird on d 1orally | Enteritidis | 14 d; 1 × 10^9 CFU/mL/bird orally | Pens; Cobb broilers | 1 d; 28 d; Ceca | n = 6, m = 7 | Acevedo-Villanueva et al., 2021 |
| Modified-live *Salmonella* Typhimurium | 1 mL 1 dose on d 1 orally | Enteritidis | 14 d; 1 × 10^9 CFU/mL/bird orally | Pens; Cobb broilers | 1 d; 28 d; Ceca | n = 6, m = 7 | Acevedo-Villanueva et al., 2021 |
| *Salmonella* Typhimurium strain UK-1 | 1 × 10^8 CFU on d 2 & 7 orally | Typhimurium | 14 d; 2 × 10^8 CFU/bird orally | Cages; Cobb 500 male broilers | 1 d; 42 d; Ceca | n = 10, m = 1 | Rubinelli et al., 2015 |
| *Salmonella* Typhimurium PBAD-mviN recombinant | 1 × 10^8 CFU on d 2 & 7 orally | Typhimurium | 14 d; 2 × 10^8 CFU/bird orally | Cages; Cobb 500 male broilers | 1 d; 42 d; Ceca | n = 10, m = 1 | Rubinelli et al., 2015 |
| *Salmonella* Typhimurium STLT2^+P13+19^ recombinant | 1 × 10^9 CFU/bird on d 1 & 7 orally | Typhimurium | 14 d; 1 × 10^9 CFU/bird orally | Cages; NA | 1 d; 28 d; Ceca | n = m = 5 | Peng et al., 2022 |
| *Salmonella* Typhimurium STLT2^+P13+19^ recombinant | 1 × 10^9 CFU/bird on d 1 & 7 orally | Enteritidis | 14 d; 1 × 10^9 CFU/bird orally | Cages; NA | 1 d; 28 d; Ceca | n = m = 5 | Peng et al., 2022 |

Abbreviations: N/A, not available; CFU, colony forming unit; PFU, plaque-forming unit; ED, embryonic day; n, experimental unit; m, measurement unit; EO, essential oil; OA, organic acid; PRO, probiotics; PRE, prebiotics; ENZ, enzyme; DFM, direct-fed microbials; dpc, day post-challenge.
